# High-light-inducible proteins control associations between chlorophyll synthase and the Photosystem II biogenesis factor Ycf39

**DOI:** 10.1093/plphys/kiaf213

**Published:** 2025-05-25

**Authors:** Anna Wysocka, Natalia Kulik, Mahendra K Shukla, Monika Opatíková, Roman Kouřil, Philip J Jackson, Amanda A Brindley, Jan Janouškovec, Éva Kiss, Andrew Hitchcock, Josef Komenda, C Neil Hunter, Roman Sobotka

**Affiliations:** Centre Algatech, Institute of Microbiology, Academy of Sciences of the Czech Republic, Třeboň 379 01, Czech Republic; Faculty of Science, University of South Bohemia, České Budějovice 370 01, Czech Republic; Centre Algatech, Institute of Microbiology, Academy of Sciences of the Czech Republic, Třeboň 379 01, Czech Republic; Centre Algatech, Institute of Microbiology, Academy of Sciences of the Czech Republic, Třeboň 379 01, Czech Republic; Department of Biophysics, Faculty of Science, Palacký University, Olomouc 779 00, Czech Republic; Department of Biophysics, Faculty of Science, Palacký University, Olomouc 779 00, Czech Republic; Plants, Photosynthesis and Soil, School of Biosciences, University of Sheffield, Sheffield S10 2TN, UK; Plants, Photosynthesis and Soil, School of Biosciences, University of Sheffield, Sheffield S10 2TN, UK; Centre Algatech, Institute of Microbiology, Academy of Sciences of the Czech Republic, Třeboň 379 01, Czech Republic; Centre Algatech, Institute of Microbiology, Academy of Sciences of the Czech Republic, Třeboň 379 01, Czech Republic; Plants, Photosynthesis and Soil, School of Biosciences, University of Sheffield, Sheffield S10 2TN, UK; Centre Algatech, Institute of Microbiology, Academy of Sciences of the Czech Republic, Třeboň 379 01, Czech Republic; Faculty of Science, University of South Bohemia, České Budějovice 370 01, Czech Republic; Plants, Photosynthesis and Soil, School of Biosciences, University of Sheffield, Sheffield S10 2TN, UK; Centre Algatech, Institute of Microbiology, Academy of Sciences of the Czech Republic, Třeboň 379 01, Czech Republic; Faculty of Science, University of South Bohemia, České Budějovice 370 01, Czech Republic

## Abstract

The biogenesis of Photosystem II is a complicated process requiring numerous auxiliary factors to assist in all steps of its assembly. The cyanobacterial protein Ycf39 forms a stress-induced complex with 2 small chlorophyll-binding, High-light-inducible proteins C and D (HliC and HliD), and has been reported to participate in the insertion of chlorophyll molecules into the central D1 subunit of Photosystem II. However, how this process is organized remains unknown. Here, we show that Ycf39 and both HliC and HliD can form distinct complexes with chlorophyll synthase (ChlG) in the model cyanobacterium *Synechocystis* sp. PCC 6803. We isolated and characterized ChlG complexes from various strains grown under different conditions and provide a mechanistic view of the docking of Ycf39 to ChlG via HliD and the structural role of HliC. In the absence of stress, chlorophyll is produced by the ChlG-HliD_2_-ChlG complex, which is stabilized by chlorophyll and zeaxanthin molecules bound to the HliD homodimer. The switch to high light leads to stress pressure and greatly elevated synthesis of HliC, resulting in the replacement of HliD homodimers with HliC-HliD heterodimers. Unlike HliD, HliC cannot interact directly with ChlG or Ycf39. Therefore, the original ChlG-HliD_2_-ChlG complex is converted into a ChlG-HliD-HliC hetero-trimer that presumably binds transiently to Ycf39 and the nascent D1 polypeptide. We speculate that this molecular machinery promotes the delivery of chlorophyll to D1 upon high-light-induced chlorophyll deficiency. The HliD homodimers formed under standard, nonstress growth conditions and attached to ChlG could serve as an emergency chlorophyll reserve.

## Teaser

The cyanobacterial High-light-inducible proteins HliC and HliD play both structural and regulatory roles in attachment of the Ycf39 assembly factor of Photosystem II to chlorophyll synthase.

## Introduction

Chlorophyll (Chl) is the central cofactor in oxygenic photosynthesis, responsible for both light capture and light-powered charge separation in Photosystems I and II (PSI, PSII). Given these essential roles, the concentration of Chl in photosynthetic membrane complexes is typically very high. However, the production and distribution of Chl is a complicated issue because free (nonquenched) Chl molecules that encounter light and oxygen can generate reactive and potentially toxic oxygen species. Since oxygen is released directly into the cell after illumination, oxygenic phototrophs must handle Chl and its porphyrin and chlorin precursors with extreme care. In contrast to carotenoids, which are also abundant cofactors in photosynthesis, the cell cannot simply keep a pool of free Chl in the membrane to support the biogenesis of the abundant Chl-binding proteins. This creates the problem of how to ensure a sufficient and balanced flux of Chl into cognate apoproteins while minimizing the level of free Chl molecules.

Tight control of Chl metabolism is particularly critical for viability under stress conditions. A change from low light (LL) to high light (HL) dramatically alters both the total requirement for Chl molecules and the channeling of Chl into individual apoproteins. In cyanobacteria, the majority (>80%) of cellular Chl is located in the trimeric PSI complex, which is also the major sink for de novo synthesized Chl molecules (Kopečná et al. 2012). However, HL-stressed cyanobacterial cells suppress PSI biogenesis (at least transiently, Hihara et al. 2001) while enhancing the synthesis of the core D1 subunit of PSII, the most frequently damaged component of the photosynthetic machinery. Thus, under stress conditions, the cell relies on a relatively small number of Chls that must be specifically channeled to D1 and, less intensively, to the other core PSII subunit, D2 (reviewed in Komenda et al. 2024). Interestingly, while the Chl-binding PSII antenna subunits CP43 and CP47 need to be preloaded with Chl molecules co-translationally as a prerequisite for their correct folding (Shen et al. 1993; Manna and Vermaas 1997), the stability of D1 and D2 is less Chl-dependent (Muller and Eichacker 1999). These 2 proteins can be detected in cyanobacterial mutants that are largely depleted of Chl (Hollingshead et al. 2016; Skotnicová et al. 2024). Chl-independent membrane insertion probably provides additional time to incorporate all Chls into D1 under stress conditions when Chl biosynthesis is downregulated.

In the model cyanobacterium *Synechocystis* sp. PCC 6803 (hereafter *Synechocystis*), 3 auxiliary PSII assembly proteins—Ycf39, Ycf48, and Pam68—have been shown to facilitate Chl insertion into PSII core subunits (Bučinská et al. 2018; Knoppová and Komenda 2019). Ycf39, a member of the atypical short-chain alcohol dehydrogenase/reductase family, is known to form a small complex with single helix High-light-inducible proteins C and D (HliC and HliD; also known as ScpB and ScpE). The Ycf39-HliD-HliC complex (hereafter 39-D/C) binds to newly-synthesized D1 and promotes D1 biogenesis in conditions of restricted Chl biosynthesis (Knoppová et al. 2014); reviewed in (Komenda et al. 2024). This 39-D/C complex remains attached to D1 after its association with the partner D2 subunit as part of the early PSII assembly intermediate named RCII* (Knoppová et al. 2022). In a *Synechocystis* mutant lacking Ycf39 and Ycf48, D1 synthesis is drastically reduced, but this process can be fully restored by enhanced Chl biosynthesis (Knoppová and Komenda 2019). It has therefore been postulated that the 39-D/C complex helps to deliver Chls to the nascent D1 and is probably also involved in the recycling of Chls released from photodamaged PSII complexes (reviewed in Komenda et al. 2024). In addition, the 39-D/C complex has the ability to dissipate excitation energy from Chls bound to RCII* (Knoppová et al. 2014); (see below). A similar, multifunctional photoprotective role is expected for HCF244-OHP1-OHP2, the homolog of the 39-D/C complex in plants and algae (Li et al. 2019; Wang et al. 2023).

Although it has been shown that the HliC/D heterodimer, rather than Ycf39, binds Chl, the mechanism by which the 39-D/C complex delivers/recycles Chl to D1 is unknown (Knoppová et al. 2014; Staleva et al. 2015). HliC and HliD (5.2 and 6.5 kDa respectively) belong to the family of High-light-inducible proteins (Hlips), which are related to the LHCs of photosynthetic eukaryotes. *Synechocystis* contains 4 Hlips (HliA-D), all of which must dimerize to form a motif for binding 4 Chls and 2 carotenoids. These pigments associate with Hlips in an energy dissipative configuration that leads to the conversion of absorbed light energy into heat (Staleva et al. 2015; Shukla et al. 2018; Konert et al. 2022 ). Through this mechanism, the excitation pressure can be released from RCII*. In contrast to Hlips, Ycf39 alone does not coordinate pigments (Knoppová et al. 2014).

The possible role of Ycf39, HliC, and HliD in cellular Chl homeostasis is strengthened by the observation that all 3 proteins were co-isolated with Chl synthase (ChlG), the terminal enzyme of the Chl biosynthesis pathway (Chidgey et al. 2014; Niedzwiedzki et al. 2016 ). A stable complex between ChlG and HliD was characterized in detail after its isolation from *Synechocystis* using FLAG-tagged ChlG (f.ChlG). In addition to Chl molecules, the ChlG-HliD complex also binds 3 different carotenoids—β-carotene (β-Car), zeaxanthin (Zea) and myxoxanthophyll (Myxo). It is noteworthy that the ChlG-HliD interaction is destabilized in the mutant lacking Zea (Proctor et al. 2020). Intriguingly, Ycf39 can be co-purified with f.ChlG and HliD from cells grown under normal light (NL) intensity but this interaction is abolished under HL (Proctor et al. 2018). HliC is another interaction partner of f.ChlG but this very small protein has so far only been detected in f.ChlG immunoprecipitations by mass spectrometry (MS), not as a stainable band on an SDS-PAGE gel as is the case for Ycf39 and HliD (Niedzwiedzki et al. 2016).

In cyanobacteria and probably also in plants, ChlG associates with Sec translocons (Chidgey et al. 2014 ; Proctor et al. 2018), which is the site where D1/D2 should be preloaded with Chl, potentially with the help of 39-D/C. However, despite numerous genetic and biochemical studies, to our knowledge there is currently no plausible model of how this machinery might work. Here, we explain the structural organization of ChlG-Hlip complexes in *Synechocystis*, including the interaction with Ycf39 and the structural role of Zea. We integrate the results of this work with those of earlier studies to propose a mechanistic model of Chl delivery to the D1 subunit of PSII via 39-D/C connected to ChlG.

## Results

### Stress-induced HliC-HliD heterodimers replace HliD homodimers in ChlG and Ycf39 complexes

**Figure 1.**
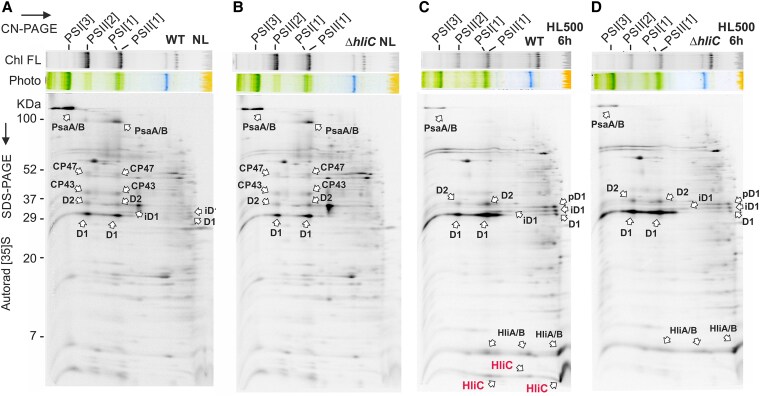
Synthesis of Hlips and the core subunits of PSI and PSII in wild type (WT) and Δ*hliC* strains. Wild-type **A** and **C)** and Δ*hliC*  **B** and **D)** cultures were grown either in NL **A** and **B)** or shifted from NL to 500 *μ*mol photons m^−2^ s^−1^ for 6 h to induce expression of Hlips **C** and **D)**. After this period cells were radiolabeled with a mixture of [^35^S]Met/Cys using a 20-min pulse at the same light intensities. Isolated membrane proteins were separated by CN PAGE with deoxycholate, with the same amounts of Chl loaded for each strain. The gel was photographed (Photo) and scanned by LAS 4000 (FUJI) for Chl fluorescence (Chl FL). Gel strips were then separated in a second dimension by SDS–PAGE. The labeled proteins were detected by a phosphorimager (Autorad). The same amounts of Chl were loaded for each strain. Proteins indicated by arrows were assigned according to (Knoppová et al. 2014; Rahimzadeh-Karvansara et al. 2022). PSI[3] and PSI[1]—trimeric and monomeric PSI; PSII[1] and PSII[2]—monomeric and dimeric PSII, respectively.

To elucidate the interactions between ChlG, Ycf39 and 2 different Hlips (HliD, HliC), we first examined the relative abundances of these proteins in *Synechocystis* grown under NL (30 *μ*mol photons m^−2^ s^−1^) or after 16 h of HL (300 *μ*mol photons m^−2^ s^−1^). Although this relatively long HL period increased the levels of ChlG, Ycf39, and HliD, these proteins are apparently constitutive components of the cell. In contrast, the HliC protein was only detectable in HL (Supplementary Fig. S1A), consistent with (He et al. 2001). During HL stress, the synthesis of HliC is very intense, as demonstrated by pulse labeling with a mixture of [^35^S]-Met/Cys (Fig. 1). After 6 h of HL, the cells were incubated with the radiolabelled amino acids, [^35^S]-Met and [^35^S]-Cys, for 20 min. After separation of membrane proteins by 2D electrophoresis, the accumulation of newly synthesized (labeled) proteins was determined by the signal of the incorporated radioisotopes. Considering that HliC contains only 2 Met and no Cys residues, while D1 contains 12 Met and 4 Cys and the radioactivity incorporation is about the same, HliC (and HliA/B) appear to be the most intensively synthesized proteins in *Synechocystis* during exposure to HL (Fig. 1, C and D).

**Figure 2.**
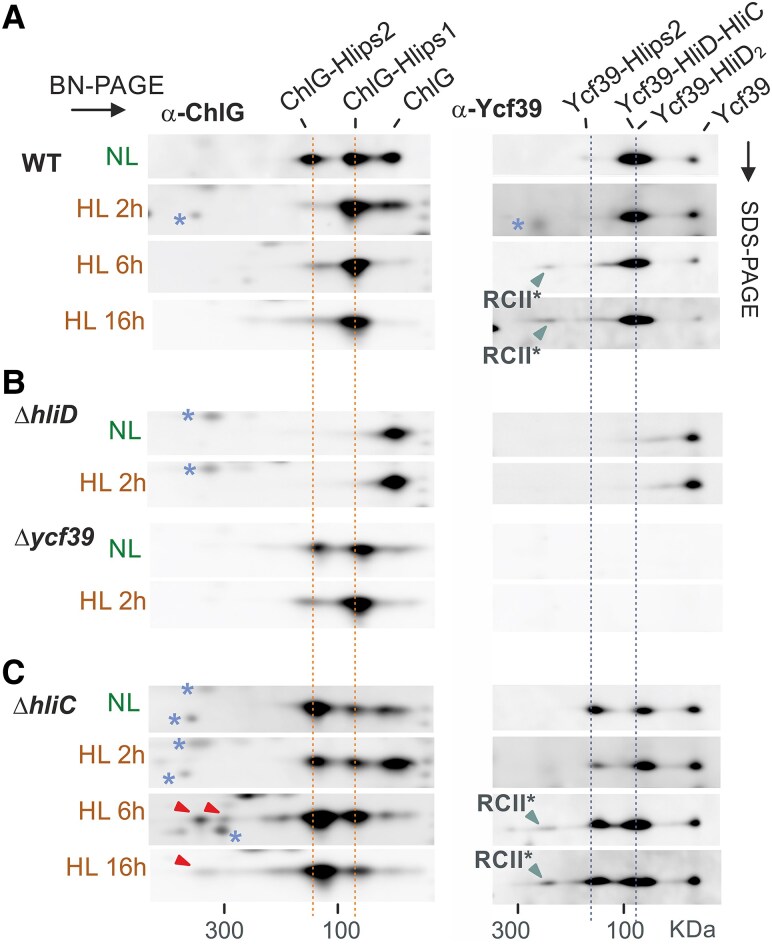
Reorganization of ChlG-Hlips and Ycf39-Hlips complexes after transition to HL. **A)**  *Synechocystis* WT cells were grown under NL conditions and then shifted to HL for 2 h, 6 h or 16 h. Membrane proteins isolated from these cell cultures were separated by 2D BN/SDS-PAGE and analysed by immunoblotting using antibodies against the ChlG and Ycf39 proteins (α-ChlG and α-Ycf39). The separate segments of the blot with individual antibody signals are shown. Dotted orange lines indicate lower- and higher-mass ChlG-Hlip1 and ChlG-Hlip2 complexes; dotted blue lines indicate 39-D_2_ (39-C/D in HL) and Ycf39-Hlip2 complexes, respectively. The signal of Ycf39 associated with RCII* is also marked by blue arrowheads. Blue asterisks indicate nonspecific cross-reactions with abundant membrane proteins. **B** and **C)** The analysis of membrane proteins described in panel **A)** was carried out using instead of WT, Δ*hliD*, Δ*ycf39*, and Δ*hliC* cells, respectively. In panel **C)**, the red arrowheads show an unknown high-mass (>300 kDa) complex containing ChlG.

After separation of *Synechocystis* membrane proteins on two-dimensional (2D) blue-native (BN)/SDS PAGE, 2 distinct complexes of ChlG with HliD can be immunodetected (Fig. 2A), previously designated as ChlG-Hlip1 and ChlG-Hlip2 (Proctor et al. 2020). Notably, we found that the larger (>100 kDa) ChlG-Hlip2 complex almost completely disappeared after 2 h at HL, and, after longer HL periods (6 and 16 h), free ChlG was barely detectable (Fig. 2A). The smaller ChlG-Hlip1 complex is thus the only ChlG form immunodetected on a 2D gel after prolonged HL treatment. We used the same samples to analyse the signal of Ycf39 and, again, at least 2 Ycf39 complexes can be distinguished. According to (Knoppová et al. 2014), the faster migrating spot represents either the 39-D/C complex or the Ycf39 attached to the HliD homodimer (39-D_2_), under high or low stress conditions, respectively. The larger Ycf39 complex, named here Ycf39-Hlips2, has not been reported previously but the absence of HliD apparently prevents its formation (Fig. 2B). Like ChlG-Hlips2, the Ycf39-Hlips2 complex also disappeared in HL (Fig. 2A). Additionally, and after 6 h of HL, a small portion of total Ycf39 can be detected bound to RCII* (Knoppová et al. 2022). We repeated this experiment by collecting the sample after 1 h of HL, which confirmed that the depletion of ChlG-Hlip2 and Ycf39-Hlips2 in HL is very rapid (Supplementary Fig. S1B).

To clarify the individual constituents of the observed ChlG and Ycf39 assemblies, we further analysed the membranes of the Δ*ycf39* and Δ*hliC* mutants. Deletion of *ycf39* has no obvious effect on the mobility of ChlG in the BN gel, although it appears to decrease the accumulation of free ChlG in NL (Fig. 2B). In the Δ*hliC* mutant grown in NL all 3 ChlG spots were clearly present, with ChlG-Hlip2 as the dominant complex. Intriguingly, all ChlG assemblies were retained in this mutant after 2 h of HL treatment and the free form of ChlG transiently became the most abundant one. Prolonged HL treatment (>6 h) almost restored the pattern of ChlG complexes to the situation in NL (Fig. 2C). In Δ*hliC*, similarly to ChlG-Hlip2, the Ycf39-Hlips2 complexes showed transient depletion and subsequent re-enrichment at the early and later phases of HL treatment, respectively. It is worth noting that the binding of Ycf39 to D1 (RCII*) was not disrupted in the absence of HliC (Fig. 2C).

**Figure 3.**
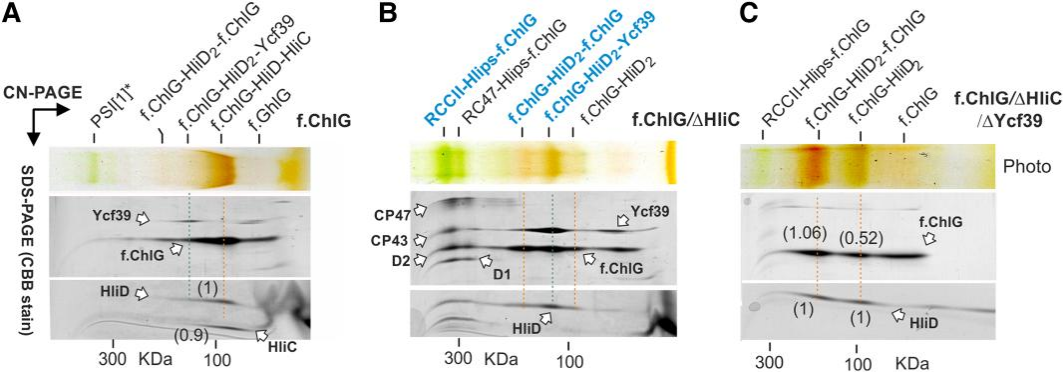
2D CN/SDS PAGE of f.ChlG complexes isolated from different genetic backgrounds. **A)** The f.ChlG enzyme was isolated from *f*.*chlG*/Δ*chlG* cells shifted to HL300 for 2 h. The obtained pulldown was resolved by CN-PAGE with A8-35 amphipol followed by SDS–PAGE in the second dimension with proteins stained by CBB stain. The separate segments of the stained gels are shown; see Supplementary Fig. S2 for full-sized gels and a control pulldown from the WT strain. The orange dashed line indicates the trimeric G-C/D complex; G-D_2_-G and G-D_2_-39 complexes are also tentatively assigned. Numbers in brackets show the molar ratio of HliC to HliD calculated from band intensities using Azure Sapphire Imager (Azure Biosystems). All proteins were identified based on previous results (Chidgey et al. 2014; Knoppová et al. 2014; Knoppová et al. 2022). **B)** Analysis of the f.ChlG pulldown obtained from the *f*.*chlG/*Δ*chlG/*Δ*hliC* strain shifted to HL for 2 h. The blue dashed line highlights the f.ChlG-D_2_-39 complex. In the absence of HliC, f.ChlG co-isolated with the PSII assembly intermediates RCCII and RC47 (Komenda et al. 2024). We propose that f.ChlG is attached to the CP47 subunit of PSII via HliA/D or HliB/D (Konert et al. 2022). Complexes marked in bold blue font were eluted from the CN gel for single-particle cryo-EM analysis (Fig. 4). **C)** CN-gel separation of the f.ChlG pulldown from a strain lacking both HliC and Ycf39; in this case the light-sensitive cells were grown under NL. Numbers in brackets show the molar ratio of HliD to f.ChlG in given complexes calculated from band intensity.

These results suggest that HliC, which accumulates during stress, plays an important role in the organization of ChlG and Ycf39 assemblies. To further investigate the effects of HliC we employed a *Synechocystis* strain that produces 3xFLAG-tagged ChlG (f.ChlG) instead of the native ChlG enzyme (*f.chlG/*Δ*chlG* strain); (Chidgey et al. 2014). This mutant was subjected to HL for 2 h and the tagged enzyme was purified with anti-FLAG resin (Koskela et al. 2020). The resulting orange eluate was separated on a clear-native (CN) PAGE with A8-35 amphipol (Kameo et al. 2021), followed by second dimension SDS-PAGE (Fig. 3A; Supplementary Fig. S2). The mobility of the main ChlG complex (∼100 KDa) corresponded to that of ChlG-Hlip1 and contained HliD and HliC in a ∼1:1 ratio (Fig. 3A). Therefore, we tentatively labeled the ChlG-Hlip1 complex as the ChlG-HliD-HliC heterotrimer (G-D/C). Two larger pigment-protein assemblies can be further resolved on the CN-gel. The large complex with the expected mobility of ChlG-Hlip2 consisted of ChlG and HliD, while another complex most likely constituted ChlG, HliD, and Ycf39 (Fig. 3A).

In a further step, we analyzed the composition of f.ChlG complexes purified from cells lacking HliC or both HliC and Ycf39. In the first case, the f.ChlG pulldown obtained from the *f*.*chlG/*Δ*chlG/*Δ*hliC* strain, the pattern of complexes obtained was more complicated than in the WT background and the f.ChlG co-isolated with a high content of Ycf39 (Fig. 3B). Based on their masses and protein composition, the two most abundant orange-colored complexes were tentatively assigned as f.ChlG-HliD_2_-f.ChlG (G-D_2_-G) and f.ChlG-HliD_2_-Ycf39 (G-D_2_-39) assemblies. These two complexes most likely correspond to the ChlG-Hlip2 and Ycf39-Hlip2 spots (Fig. 2), whose mobility on the BN gel appears identical for the WT, whereas two distinct signals are seen in the f.ChlG strains due to the presence of the tag (Supplementary Fig. S3). An additional weaker orange band in the ChlG pulldown sample obtained from the *f*.*chlG/*Δ*chlG/*Δ*hliC* strain was assigned as f.ChlG-HliD_2_ (G-D_2_; Fig. 3B).

Two green complexes containing PSII core subunits also co-isolated with f.ChlG in the absence of HliC (Fig. 3B). As recently reported (Konert et al. 2022 ), HliD can bind the HliA and HliB Hlips in the absence of HliC, their genuine partner. During HL stress, HliA/C and HliB/C heterodimers are associated with the PSII assembly intermediates RCCII (PSII core complex) and RC47 (CP43-less PSII intermediate; Komenda et al. 2024). Replacement of HliC by HliD could therefore lead to aberrant attachment of ChlG to assembling PSII via HliA-HliD or HliB-HliD pairs (see below). The probable signals of ChlG associated with RCCII via Hlips are highlighted by red arrowheads on the immunoblot following separation in the second dimension in Fig. 2C.

The additional elimination of Ycf39 simplified the range of the isolated f.ChlG complexes, and only G-D_2_-G and G-D_2_ can be detected together with the green RCCII-Hlips-ChlG complex. This simple pattern of Coomassie-stained proteins enabled us to quantify the molar ratio of f.ChlG and HliD in the gel, by taking into account the staining intensities and the respective molecular masses of f.ChlG and HliD. The molar ratios calculated for G-D_2_-G and G-D_2_ were ∼1:1 and ∼1:2, respectively, in agreement with our predictions.

The data shown so far imply that the HliD dimers associated with ChlG and Ycf39 in NL are replaced by HliC/D heterodimers in HL and the larger G-D_2_-G complex is disassembled. To further verify the composition of the ChlG complexes using an alternative (“gel-free”) approach, we determined the molar quantities of f.ChlG, HliD, and Ycf39 in the f.ChlG pulldowns using quantitative MS with ^15^N-labeled internal standards (Supplementary Table S1 and Supplementary Data Set 1). As shown in Table 1, the f.ChlG pulldown isolated from NL–grown cells contained f.ChlG and HliD proteins in a ratio of about 1:1.5, which would agree with a mixture of G-D_2_-G and G-D_2_ complexes. After 2 h of HL, the ratio between f.ChlG and HliD decreased to 1:1.1, but only when HliC was present; in the Δ*hliC* background the ratio remained close to 1:1.5 (Table 1). These numbers agreed with our prediction that HliC is recruited to ChlG complexes as a HliC-HliD heterodimer. Moreover, quantitative MS confirmed the marked effect of HliC in suppressing the stable interaction between Ycf39 and f.ChlG during HL; the ratio of these proteins in the f.ChlG pulldown approached 0.7 Ycf39 per ChlG after elimination of HliC (Table 1), consistent with the result of the 2D CN/SDS PAGE (Fig. 2, A and B).

**Table 1.** Quantification of f.ChlG, HliD, and Ycf39 proteins in f.ChlG pulldowns

Experiment	Protein	Quantity (pmols ± SD) in pulldown (total volume)	Stoichiometry (ratio per 1 ChlG ± propagated SD)
f.ChlG NL	f.ChlG	60.33 ± 0.14	1
	HliD	92.79 ± 0.74	1.54 ± 0.013
	Ycf39	2.58 ± 0.47	0.04 ± 0.008
f.ChlG/ΔHliC NL	f.ChlG	37.19 ± 0.96	1
	HliD	54.74 ± 3.15	1.47 ± 0.093
	Ycf39	2.02 ± 0.34	0.05 ± 0.009
f.ChlG HL 16 h	f.ChlG	32.06 ± 0.59	1
	HliD	35.96 ± 3.14	1.12 ± 0.010
	Ycf39	0.12 ± 0.02	0.004 ± 0.001
f.ChlG/ΔHliC HL 16h	f.ChlG	6.92 ± 0.07	1
	HliD	10.46 ± 0.78	1.51 ± 0.11
	Ycf39	4.98 ± 1.55	0.72 ± 0.22

*Synechocystis* cells expressing f.ChlG in a Δ*chlG* background, and with the additional deletion of *hliC*, were grown under NL and HL for 16 h. Proteins were quantified as described in Supplementary Data Set 1 and (Pratt et al. 2006) using ^15^N-labeled internal standards (Supplementary Table S1).

### Structural determinants mediating the interaction between ChlG, Ycf39, and Hlips

**Figure 4.**
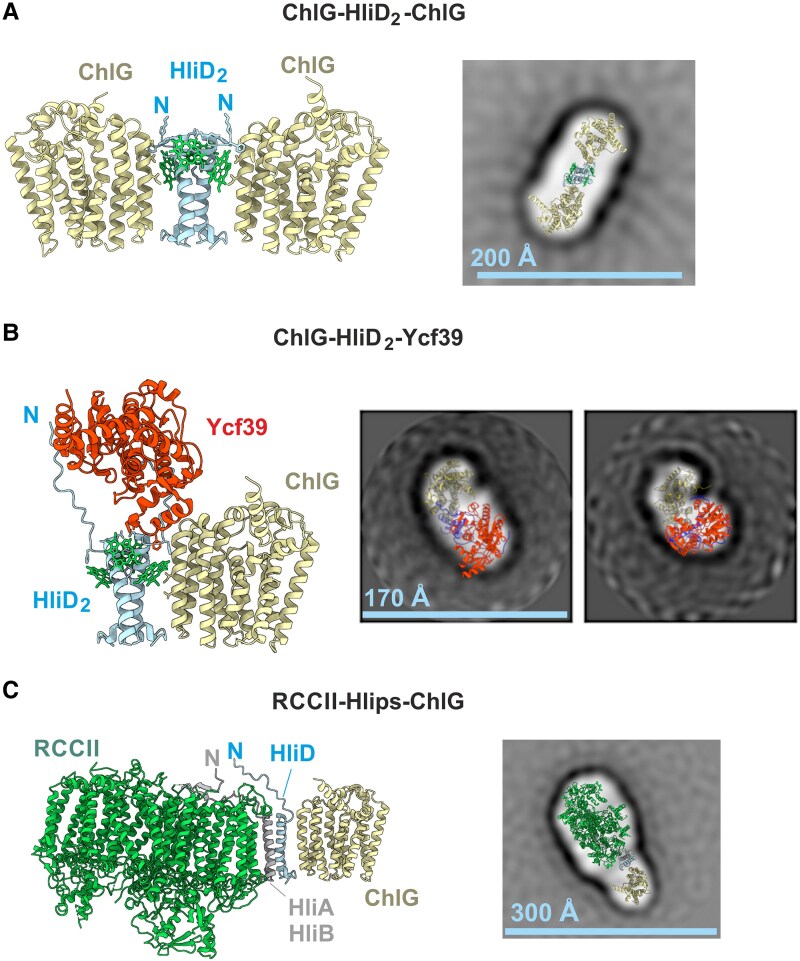
*S*ingle particle electron microscopy of negatively stained protein complexes isolated using f.ChlG from different *Synechocystis* mutants. **A)** AlphaFold3 prediction of the G-D_2_-G complex with 4 Chls and a fit of this model to the averaged projection map from single-particle EM analysis (see Supplementary Fig. S5 for the gallery of classified single particle classes). This protein complex was eluted from a CN gel as indicated in Fig. 3B. The N-terminal ends of Hlips are indicated. **B)** Structural analysis of the negatively stained G-D_2_-39 complex. **C)** Structural analysis of the negatively stained RCCII complex with attached f.ChlG-Hlips (HliA/D or HliB/D). As indicated by MD simulations, the attachment of Ycf39 to HliD is rather flexible (Supplementary Fig. S7), which is in line with the obtained range of classes in the single-particle analysis (Supplementary Fig. S6). Only the projections for the 2 most frequent classes are shown in **B)**, see Supplementary Fig. S8 for more classes. The spectrum of classes for the RCCII-Hlip-f.ChlG complex is shown in Supplementary Fig. S9.

Knowledge of the stoichiometry of the individual protein complexes allowed us to model their structures using AlphaFold3 (Abramson et al. 2024). The models containing Hlips also included 4 Chls, based on the presence of 4 conserved Chl-binding motifs for a Hlip dimer (Shukla et al. 2018). We obtained statistically reliable predictions for all of the ChlG complexes described above (see Supplementary Fig. S4). ChlG binds the HliD homodimer or the HliC-HliD heterodimer laterally (via HliD), whereas the Ycf39 does not interact directly with ChlG but binds the N-terminal segments of both HliDs in G-D_2_ (Fig. 4B; Supplementary Fig. S4B). The binding of Chl to Hlips in all AlphaFold3 models (Fig. 4, Supplementary Fig. S4) corresponded to our previous prediction, which was based on the organization of Chl cofactors in LHC antennae (Shukla et al. 2018).

The relatively larger masses of the G-D_2_-G and G-D_2_-39 assemblies allowed us to compare the predicted structural models with the shape of the complexes visualized by single particle electron microscopy (EM) of negatively stained specimens. Both complexes were eluted from the CN gel (f.ChlG pulldown obtained from the *f*.*chlG/*Δ*chlG/*Δ*hliC* strain, see Fig. 3B) and subjected to single particle analysis. According to the AlphaFold3 prediction (Fig. 4A, Supplementary Fig. S4), the G-D_2_-G complex is expected to have an elongated, symmetrical shape. This prediction is in good agreement with the most abundant class of particles (Supplementary Fig. S5) observed in the single-particle analysis (Fig. 4A). The classification of G-D_2_-39 particles was more complicated (Supplementary Fig. S6), indicating the presence of a flexible component in the complex. Since Ycf39, which is attached to the N terminus of HliD, is relatively free to move, we utilized molecular dynamics (MD; Supplementary Fig. S7 and see also below) to assign the predicted complex to the most common classes (Supplementary Fig. S8). We were able to identify projected structural conformations of G-D_2_-39 particles that fit well into 2 different classes of G-D_2_-39 particles (Fig. 4B), thus the single-particle EM analysis agrees with the AlphaFold3 predictions and supports the stoichiometry of the individual proteins.

The putative RCCII-Hlips-ChlG assembly was quite abundant in the f.ChlG pulldown obtained from the *f*.*chlG/*Δ*chlG/*Δ*hliC* strain and we therefore decided to analyze this large complex also by EM. Single particle EM analysis revealed very homogeneous particles (Supplementary Fig. S9), and the projection of the AlphaFold3 prediction (Fig. 4C) is consistent with ChlG being attached to the CP47 site of RCCII via Hlips, in agreement with our previous work (Konert et al. 2022).

**Figure 5.**
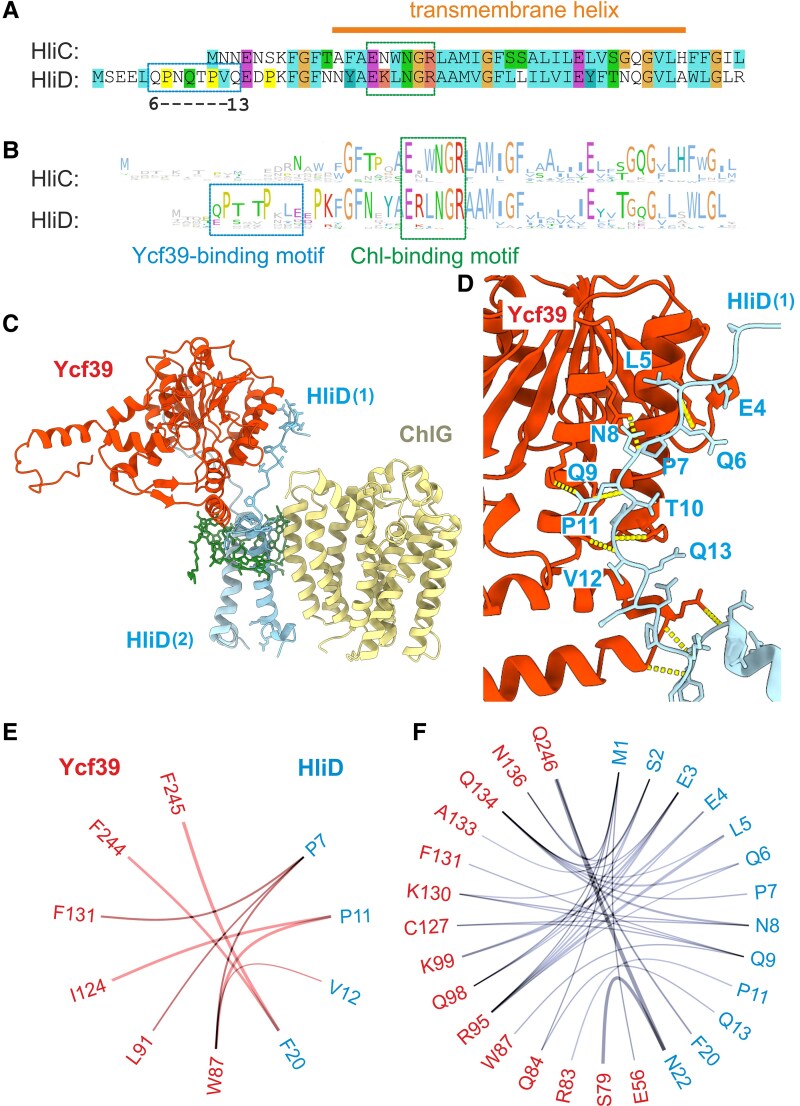
A conserved motif in the HliD N-terminus is important for the binding of Ycf39 to HliD. **A)** Aligned amino acid sequence of *Synechocystis* HliC and HliD proteins; the putative Ycf39-binding motif in HliD (residues 6 to 13) is outlined by a blue box, and the Chl-binding motif present in both proteins is highlighted by a green box. **B)** Sequence logo of the HliC and HliD proteins from genomes of 37 phylogenetically representative cyanobacterial species created in Jalview (Waterhouse et al. 2009), based on an alignment of HliC and HliD sequences covering all known phylogenetic groups of cyanobacteria. **C)** A final configuration from the MD simulation of the G-D_2_-39 complex after 1 µs (run 1, see Supplementary Fig. S7 for details) showing the interaction between Ycf39 and the N-terminus of HliD(1). **D)** Close-up view of the interaction between Ycf39 and HliD(1) showing predicted hydrogen bonds (yellow dashed lines) between Ycf39 and residues of the HliD N-terminal motif. **E** and **F)** Flare plots showing frequencies of molecular interactions between Ycf39 (red letters) and HliD(1) (blue letters) across MD simulation frames during period of MD (200 to 1000 ns), run 1 (Supplementary Fig. S7). Hydrophobic interactions and hydrogen bonds are depicted in plots **E** and **F)**, respectively.

The structural model of the G-D_2_-39 complex (Fig. 4B) predicts that the Ycf39 binds to a conserved motif in the N-terminus of HliD, which is absent in the shorter HliC protein (Fig. 5A). If this model is correct, the Ycf39 binding motif should be conserved in HliD proteins from different species. To clarify this point, we constructed a sequence logo of HliC and HliD proteins based on 37 genomes of cyanobacteria sampled across their evolutionary diversity (see Materials and methods). This analysis revealed that HliD contains a highly-conserved N-terminal PTxTP motif, which is absent from HliC (Fig. 5B).

To gain deeper insight into the interactions between Ycf39, HliD, and ChlG, we performed 3 independent 1-μs MD simulations that were based on the AlphaFold3-predicted G-D2-39 complex embedded in the thylakoid membrane (Supplementary Videos S1 and S2). The calculation showed that structure of ChlG is equilibrated after ∼120 ns, while the position of Ycf39 fluctuates substantially in respect to ChlG, indicating its flexibility within the complex (Supplementary Fig. S7A). From the root mean square deviation (RMSD) values and hydrogen bond analysis we confirmed that Ycf39 is not rigidly bound to ChlG. At the beginning of the MD simulation, the Ycf39 preferentially formed hydrogen bonds with the HliD(1) protein that is in contact with ChlG (Fig. 5, C and D; Supplementary Fig. S7B, green line). Although Ycf39 also formed hydrogen bonds with ChlG and the second copy of HliD during the course of the MD simulation, these interactions were transient. Moreover, the interaction between Ycf39 and HliD(2) was completely lost in 1 of 3 runs, while Ycf39 became attached to the complex only through HliD(1) (Supplementary Fig. S7B). According to the MD, the stable attachment of Ycf39 to HliD(1) is provided by a network of interactions between the HliD N-terminus and a pair of alpha-helices of Ycf39 (Fig. 5, E and F). Indeed, the conserved prolines in the PTxTP motif is modified to ^7^PNxTP^11^ in HliD and play an important role in binding of Ycf39 and HliD(1), contributing to both hydrophobic and hydrophilic interactions (Fig. 5, E and F). Residue N^8^ of Ycf39 interacts with HliD(1) by backbone atoms, hence this interaction will be preserved in homologs with an N to T substitution.

**Figure 6.**
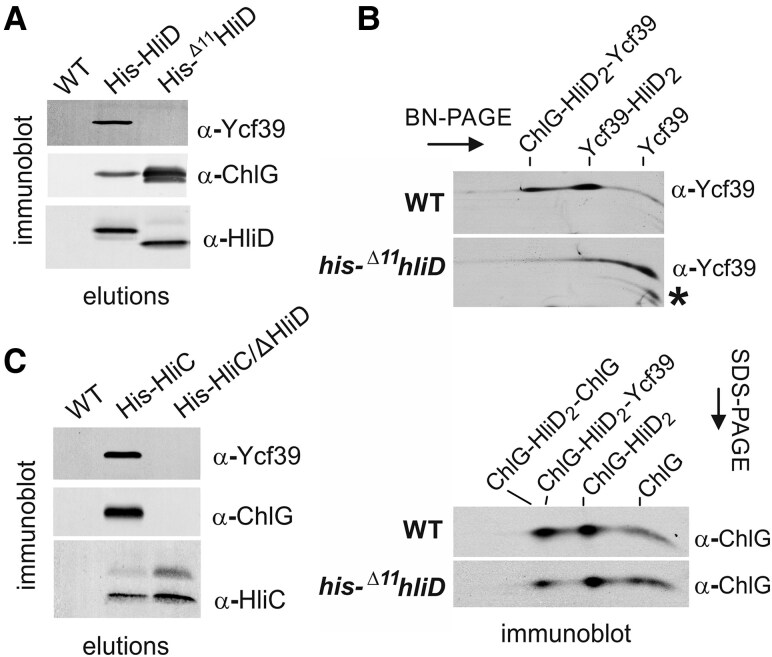
Interaction of His-tagged HliD, ^Δ11^HliD and HliC with Ycf39 and ChlG. **A)** The His-HliD and His-^Δ11^HliD proteins were isolated from HL-treated cells (2 h) using nickel-affinity chromatography together with a WT control elution. The levels of ChlG, HliD, and Ycf39 in the eluates were assessed by immunodetection after separation by SDS–PAGE. 1/20 of the total elution volume was loaded into each lane. **B)** Membrane proteins, isolated from WT and *his*-^Δ11^*hliD* cells grown under NL, were separated by 2D BN/SDS–PAGE and analyzed by immunoblotting with ChlG and Ycf39 antibodies. Protein complexes are marked. The separate segments of the blot with individual antibody signals are shown. The asterisk marks a Ycf39 degradation product. **C)** Isolation of His-HliC, produced in a genetic background lacking the native *hliC* gene and from a strain with additional deletion of the *hliD* gene. All strains including the WT control were grown under HL for 2 h.

The role of the HliD N-terminal motif in the interaction between Ycf39 and ChlG was verified biochemically using *Synechocystis* strains expressing His-tagged HliD or a truncated His-^Δ11^HliD variant (lacking the first 11 residues) in place of the native *hliD* gene in the genome. We isolated both proteins from HL-treated cells (2 h) using nickel-affinity chromatography and analyzed the levels of co-isolated Ycf39 and ChlG by immunodetection. Although ChlG was present in both isolations, Ycf39 was only eluted together with the full-length HliD, but not together with the truncated His-^Δ11^HliD (Fig. 6A). The loss of interaction between Ycf39 and His-^Δ11^HliD (but not between ChlG and His-^Δ11^HliD) was confirmed by 2D BN/SDS-PAGE (Fig. 6B). These data confirm our *in silico* prediction (Fig. 5) that the HliD N-terminus is essential for the stable binding of Ycf39 to HliD but it is not required for the interaction between HliD and ChlG.

To clarify the role of HliC in the formation of ChlG complexes, we used a previously constructed *his*-*hliC*/Δ*hliC* strain that expresses the *his*-*hliC* gene from the constitutive *psbAII* promoter and lacks the native *hliC* gene (Shukla et al. 2018). As expected, His-HliC was eluted along with ChlG and Ycf39, presumably by binding to HliD, which acts as a linker (Fig. 6C). In order to exclude any direct interaction between HliC and ChlG/Ycf39, we deleted the *hliD* gene in the *his*-*hliC*/Δ*hliC* background and in this case His-HliC was isolated without additional partners, consistent with our structural models (Supplementary Fig. S4). We can therefore conclude that HliC does not directly associate with Ycf39 and ChlG, and the interaction depends solely on HliD.

**Figure 7.**
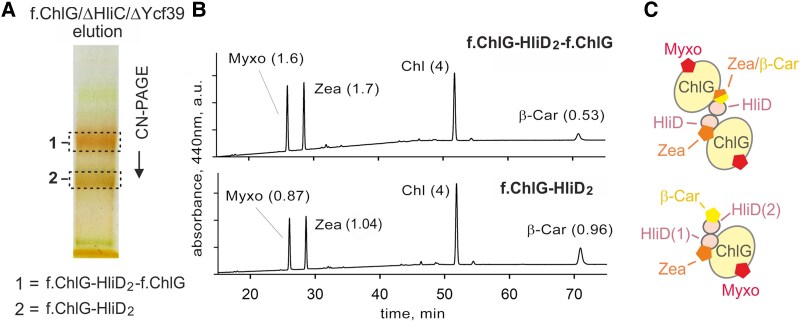
Stoichiometry of pigments associated with f.ChlG-HliD complexes. **A)** f.ChlG-HliD complexes were isolated from the *Synechocystis f*.*chlG/*Δ*chlG/*Δ*hliC*/Δ*ycf39* strain grown at NL. The eluate obtained was separated by CN-PAGE and the protein bands, indicated by dashed boxes, were excised from the gel. The upper band (1) represents the G-D_2_-G complex, and the lower band (2) is the G-D_2_ complex (see Fig. 3C). **B)** Assessment of the pigment composition of the G-D_2_-G and G-D_2_ complexes shown in panel **A)**. Pigments were extracted from the complexes and quantified by HPLC; the relative amounts of carotenoids were calculated per 4 Chls (values in parentheses). **C)** A model showing the predicted association of carotenoids with the G-D_2_-G and G-D_2_ complexes. In the tetrameric complex, the ratio of 0.5 β-Car per 4 Chls suggests that Zea preferentially binds at the HliD-ChlG interface. Myxo is most likely a peripheral carotenoid that interacts only with ChlG (see Discussion). In the trimeric complex, β-Car binds the HliD(2) copy, which we predict is not in direct contact with ChlG.

ChlG-Hlips complexes are pigment-protein assemblies that bind Chl and a unique mixture of the carotenoids β-Car, Zea and Myxo (Chidgey et al. 2014). Puzzlingly, Zea has been shown to stabilize the interaction between ChlG and HliD (Proctor et al. 2020) but free Hlips, including dimeric HliC and the 39-D_2_ complex, have so far been purified exclusively with β-Car (Staleva et al. 2015 ; Shukla et al. 2018; Knoppová et al. 2022 ). To elucidate the structural role of Zea, we eluted the purified and gel-separated G-D_2_-G and G-D_2_ assemblies from the CN gel (Fig. 7A) and analyzed the extracted pigments by HPLC (Fig. 7B). The stoichiometry of each carotenoid species was normalized to 4 Chls, which represents the expected number per Hlip pair together with 2 carotenoids (Shukla et al. 2018; Konert et al. 2022 ). Interestingly, we found only about 0.5 β-Car per 4 Chls in the G-D_2_-G complex but 1.7 and 1.6 of Zea and Myxo, respectively (Fig. 7B). It is therefore likely that the HliD homodimer preferentially binds a xanthophyll when docked to ChlG. As will be explained later, it is reasonable to expect that Zea interacts with both HliD and ChlG, thereby stabilizing the complex, while Myxo binds peripherally to ChlG (Fig. 7C). Compared with G-D_2_-G, the G-D_2_ complex was less orange because it binds only 3 carotenoids (β-Car: Zea: Myxo 1:1:1) per 4 Chls, in agreement with the model presented in Fig. 7C.

## Discussion

Although definitive evidence is still lacking, it is widely accepted that Chl biosynthesis in cyanobacteria and chloroplasts takes place in specific “biogenic” membrane domains alongside the translation of the protein subunits of assembling photosystems (Ostermeier et al. 2024). In these biogenesis zones, the translating polypeptides are inserted into the lipid bilayer, where they are loaded with Chl and other pigments and cofactors and assembled into functional complexes. This process is highly organized, supported by many auxiliary proteins and synchronized with other processes in the cell (see Komenda et al. 2024; Ostermeier et al. 2024 for recent reviews). ChlG, which catalyzes one the final steps of Chl biosynthesis (Proctor et al. 2020), is associated with the YidC insertase, a component of the protein translocation system (Chidgey et al. 2014). Chl is most likely released from ChlG in close proximity to the nascent chains of Chl-binding proteins, which may be fixed by YidC and Ycf48 in a conformation to facilitate Chl insertion (Yu et al. 2018). Thus, ChlG and the insertase are believed to cooperate in completing the synthesis of Chl synthesis, and delivering it to nascent Chl-binding membrane proteins, enabling their correct folding and assembly in the thylakoids (Chidgey et al. 2014).

Despite the close physical and mechanistic linkage between the production of Chl and its binding to newly-synthesised cognate proteins, some unattached Chl molecules could accumulate in the membrane. However, a limited pool of free Chl is unlikely to be a major problem for the cell if it is surrounded by carotenoids. For cells growing under LL/NL, which preferentially directs Chl to the PsaA and PsaB subunits of PSI, such a pool could even be vital (Kopečná et al. 2012). Each PSI core protein binds about 45 Chls and has thus a capacity to scavenge free Chl from the biogenesis membrane. A certain availability of free Chl could therefore facilitate the biogenesis of PsaA/B as well as the Chl-demanding subunits of PSII (CP43 and CP47), as has been shown for a strain with mutated variant of CP47 (Sobotka et al. 2005). However, leaky and less precise Chl delivery relying on free Chl in the membrane is unlikely to be acceptable under harsh conditions. The supply of de novo Chl can be drastically restricted during stress (Hihara et al. 2001; Chen et al. 2021), and the remaining Chl should be targeted to D1 and D2 subunits to maintain PSII functionality. Apart from newly synthesized Chl, cells can also reuse Chl released from degraded photosystems (Vavilin et al. 2007).

**Figure 8.**
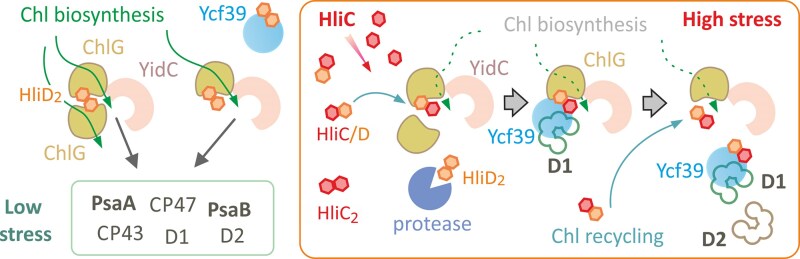
A working model of the Hlip-mediated associations between Ycf39 and ChlG in response to stress conditions. Under low stress, Chl molecules are largely produced de novo and incorporated mainly into the nascent PsaA/B subunits of PSI (Kopečná et al. 2012). ChlG complexes are organized as ChlG-HliD tetramers or trimers. A higher concentration of ChlG, associated with a single translocon (Chidgey et al. 2014) could facilitate fast loading of Chl cofactors into Chl-rich proteins such as PsaA/B or the antenna subunits of PSII. Ycf39 is bound to dimeric HliD and probably does not play a major role. Under stress conditions, the synthesis of PSI subunits and the Chl pathway are strongly suppressed (Hihara et al. 2001; Kopečná et al. 2012) but synthesis of the core D1 and D2 subunits of PSII must continue to maintain PSII activity (see Fig. 1). To facilitate D1 biogenesis, HliC accumulates, and the newly formed HliC/D heterodimers bind to ChlG complexes. However, unlike HliD, HliC does not directly interact with ChlG (and Ycf39). Therefore, an HliC/D pair binds just a single ChlG, leading to the disappearance of G-D_2_-G tetramers and formation of G-D/C trimers. We speculate that Ycf39 still (transiently) contacts the G-D/C but the attachment of the nascent D1 chain to Ycf39 dissociates the D1-39-D/C subcomplex from ChlG. After disruption of the ChlG-HliD interaction, Chl molecules bound to HliD could be released from the complex together with a Zea. This released Chl can then bind to D1 to support the assembly of the RCII core. Unbound Hlip pairs can also bind either Chl or chlorophyllide from degraded photosystems and transfer it back to ChlG and Ycf39, thus participating in the recycling of Chl (Vavilin et al. 2007).

The above considerations emphasize the need to have small, mobile Chl-binding proteins that can act as intermediaries between the enzymes producing Chls, and the machinery for assembling photosystems from their pigment and protein components. These proteins, Hlips, can help to resolve problems that could arise under HL conditions, and they play an essential role in photoprotection in cyanobacteria. When grown under 500 *μ*mol photons m^−2^ s^−1^ the *Synechocystis* Δ*hliC*/Δ*hliD* double mutant was rapidly outcompeted by the WT (He et al. 2001) and a strain lacking all 4 Hlips was no longer viable at this light intensity (Havaux et al. 2003). According to various lines of evidence, Hlips are involved in: (i) scavenging and quenching of free Chl (Sinha et al. 2012); (ii) recycling of Chl molecules released from degraded Chl-binding proteins (Vavilin et al. 2007); (iii) delivery of Chl to the D1 subunit of PSII (Knoppová et al. 2014); and (iv) quenching of Chl associated with PSII assembly intermediates (Knoppová et al. 2014, 2022). It is clear that Hlips are vital for managing fluctuations in the provision of Chls, in buffering their availability in the membrane bilayer, and finally in interfacing with the many Chl-binding proteins that can become available as a result of biosynthesis and turnover. At the same time, they must also bind carotenoids to prevent the damaging consequences of Chls absorbing light. It is unclear how Hlips could fulfill all these tasks, but our demonstration of ChlG-Hlip complexes that adapt to light stress does allow us to propose a model for their function (Fig. 8).

All 4 *Synechocystis* Hlips bind Chl and carotenoid molecules in a very efficient energy-dissipative configuration (Staleva et al. 2015; Shukla et al. 2018; Konert et al. 2022). Although clear evidence is missing, the phototoxicity of free Chls thus could be eliminated just by accumulation of Hlips in the membrane. However, other tasks require interactions with a subset of protein partners including other Hlips. HliA/C and HliB/C pairs, which were proposed to dissipate absorbed energy from the CP47 assembly module as well as from RC47/RCCII (Konert et al. 2022). The interaction occurs via the N-terminus of HliA/B, which binds specifically to the CP47 assembly module (Promnares et al. 2006; Bučinská et al. 2018; Konert et al. 2022). HliD can accumulate as a homodimer (in LL/NL) or, upon stress, as a HliD/C heterodimer (Staleva et al. 2015). As we show here, the helical part of HliD associates with ChlG, while a conserved motif in the N-terminus binds Ycf39 (Fig. 5). In the absence of HliC, HliD can bind the stress-induced HliA/B (Konert et al. 2022), which results in aberrant structures that physically link PSII assembly complexes to ChlG (Fig. 4).

An interesting finding of our work is the structural role played by pigments in the interaction of HliD with ChlG. Although dissociation of ChlG-Hlips complexes in the absence of Zea has been described by (Proctor et al. 2020), the location of Zea was enigmatic. Here, we analyzed pigments extracted from isolated G-D_2_-G and G-D_2_ complexes (Fig. 7), rather than from a mixture of isolated ChlG complexes as in previous reports (Chidgey et al. 2014; Niedzwiedzki et al. 2016). This approach has revealed that Zea is the preferred carotenoid in G-D_2_-G complexes, which are sufficiently stable for isolation via a FLAG-tag and on CN-PAGE. It seems likely that ChlG creates a hydrophilic cavity that preferentially accommodates the Zea β-ring with its hydroxyl group rather than the hydrophobic β-Car. Although *Synechocystis* strains lacking Zea still contain some G-D_2_ complex with β-Car and Myxo, most of the HliD dissociates from f.ChlG during purification (Proctor et al. 2020). Zea thus provides greater stability for G-D_2_ than β-Car. On the other hand, the absence of Myxo had no effect on the stability of the complex (Proctor et al. 2020) and, as (Niedzwiedzki et al. 2016) has shown, Zea (and β-Car), but not Myxo, are involved in the quenching of Chl in G-D/C complexes. We therefore expect Myxo binds peripherally to ChlG and does not interact directly with Hlips (Fig. 7).

HliD is the only constitutively produced Hlip in *Synechocystis*, and, under NL, is distributed as a homodimer in complexes with either ChlG or Ycf39. Based on quantitative MS, the 39-D_2_ complex should be about 2-fold more abundant than G-D_2_(-G) (Jackson et al. 2023). It is difficult to judge what fraction of ChlG occurs in vivo in larger structures (G-D_2_-G) but, based on our MS data, it should be at least half (Table 1). As discussed above, these complexes probably partially dissociate on native gels or during purification if they contain β-Car. The amount of G-D_2_-39 in WT might also be greater than we observe on a BN gel (Fig. 2A) but is presumably still very low.

We have combined the results of this study with those of previous publications to propose a model for the Hlips-driven associations between Ycf39, ChlG and possibly the D1 subunit of PSII (Fig. 8). In *Synechocystis* cells acclimated to low stress conditions (e.g. NL, 28 °C), more than 80% of de novo synthesized Chl is dedicated for incorporation into PSI (Kopečná et al. 2012). At least some Chls must be co-translationally inserted into PsaA/B (and also into CP43/CP47) as a prerequisite for the correct folding of these proteins (Klein et al. 1988; Manna and Vermaas 1997). Oligomerization of ChlG via HliD could therefore be advantageous to ensure sufficient Chl supply to a single translocon system. ChlG accumulates significantly less in the Δ*hliD* mutant (Chidgey et al. 2014), suggesting that the binding of HliD is important for the stability of ChlG and “free” ChlG is degraded by the cellular proteolytic machinery.

Under less favorable conditions, de novo Chl biosynthesis is restricted (Hihara et al. 2001; Kopečná et al. 2012; Chen et al. 2021) and the cell intensively produces the short-lived D1/D2 subunits (Fig. 1), also relying on Chl released from degraded Chl-binding proteins (Vavilin et al. 2007; Hollingshead et al. 2016). HliC also accumulates massively under stress conditions (Fig. 1), forming homodimers (Shukla et al. 2018) and heterodimers with other Hlips (Konert et al. 2022). As we demonstrated here, HliC_2_ pairs do not bind to ChlG or Ycf39 (Fig. 6C), although they have capacity to scavenge or quench free Chl in membranes (Shukla et al. 2018). However, HliC/D dimers form complexes with ChlG and Ycf39 and we suggest that they fulfill additional tasks. First, the HliC/D heterodimer replaces HliD_2_ homodimers bound to ChlG and Ycf39. This process is very fast (Supplementary Fig. S1B), suggesting that the HliD_2_ dimers are targeted for degradation during stress, most probably by the FtsH4 complex (Krynická et al. 2023). HliC, with its short N-terminus, is expected to be a much poorer substrate for the protease than HliD. We speculate that ChlG-associated HliD_2_ serves as an emergency reserve of Chl molecules, released after the shift into stress to support PSII repair.

Since HliC does not interact directly with ChlG (Fig. 6C), G-D_2_-G complexes are converted to G-D/C after the induction of HliC synthesis (Table 1). Unexpectedly, we could not isolate a hypothetical G-D/C-39 complex from HL-treated cells, although Ycf39 tightly binds the HliD/C pair (Knoppová et al. 2014), see also (Supplementary Fig. S10). According to our predictions and MD analysis, the N-terminus of HliD is not involved in attachment to ChlG and it should be available to bind Ycf39 (Fig. 5). Moreover, the Ycf39 also interacts with the G-D_2_ complex, implying that the presence of ChlG does not interfere with Ycf39 binding. One possible explanation is that the attachment of Ycf39 specifically to the HliD(1) copy, as suggested by our AlphaFold3 modeling (Fig. 5; Supplementary Fig. S4), makes the G-D_2_-39 complex unstable and impossible to purify or isolate by native PAGE. The stable G-D_2_-39 variant that accumulates in the Δ*hliC* mutant (Fig. 2C), might have Ycf39 bound to the HliD(2) copy that is not in direct contact with ChlG. This complex may thus represent just another aberrant assembly complex such as RCCII-Hlips-ChlG. This would also explain why we cannot detect the interaction of Ycf39 with the G-D_2_-G complex because, after Ycf39 attachment to HliD, the adjoining copy of ChlG is released.

In our working model (Fig. 8) we speculate that Ycf39 binds to the G-D/C complex during stress and that this complex functions primarily in the biogenesis of D1/RCII and Chl recycling. It is notable that f.Ycf39 produced in a *Synechocystis* D1-less strain purifies with a relatively high ChlG content, while no ChlG was detected in f.Ycf39 preparations from other genetic backgrounds (e.g. D2-less; Knoppová et al. 2014). Based on these data, we propose a working model that, when the nascent D1 chain exits the SecY/YidC translocon, it attaches to Ycf39, which sits nearby attached to G-D/C. The Ycf39 then dissociates HliD from ChlG, releasing Zea because of its tight contact with ChlG. In fact, we have never detected the 39-D/C complex with Zea but only with β-Car (Staleva et al. 2015; Knoppová et al. 2022), even when it was dissociated from f.ChlG during size exclusion chromatography (Niedzwiedzki et al. 2016). Without Zea, the binding of Chls to HliD is probably destabilized (Skotnicová et al. 2021) and the released Chls can immediately insert into D1. The remaining Chls and β-Car at the HliC site could be still able to quench the formed RCII*. This hypothesis is in line with only 1 additional β-Car present in the quenched RCII* complex when the content of pigments is compared with the unquenched RCII (Knoppová et al. 2022). In our model (Fig. 8) the “free” ChlG is ready to accept another HliD/C pair containing either Chl or chlorophyllide collected from damaged complexes (Vavilin et al. 2007). This mechanism ensures that Chl is precisely delivered to D1, that the early PSII assembly step is protected, and that the free Chl is safely redirected back to the translocon machinery (Fig. 8).

The conserved eukaryotic proteins OHP1 (One Helix Protein 1) and OHP2 are similar to cyanobacterial Hlips. Like HliC and HliD, OHP1 and OHP2 form a heterodimer (Hey and Grimm 2018). Furthermore, OHP1 and OHP2 are both HL-inducible but not functionally redundant, as elimination of OHP1 or OHP2 in plants and green algae leads to defective synthesis of D1 (Beck et al. 2017; Li et al. 2019; Wang et al. 2023). The OHP1/2 hetero-oligomer associates with PSII RC proteins (Maeda et al. 2022), and a direct interaction between the Ycf39 homolog HCF244 and OHP2 has also been demonstrated (Hey and Grimm 2018; Li et al. 2019). Based on these similarities, OHP1/2 is likely to fulfill the same physiological function as HliC/D. We hypothesize that the formation of the OHP1/2 heterodimer facilitates the feeding of Chl into the PSII machinery through controlled interactions between the HCF244 factor and RCII complexes, analogous to cyanobacterial HliC/D and Ycf39. A structural model of the Arabidopsis HCF244-OHP1/2 is shown in Supplementary Fig. S10; note the striking similarity to the 39-D/C complex.

HCF244 is essential for the initiation of translation of *psbA* mRNA (Link et al. 2012), a highly dynamic process that controls the rate of D1 synthesis in the plastid (Chotewutmontri and Barkan 2018). It is not known whether Ycf39 plays the same role in translation initiation in cyanobacteria, but given the weak phenotype of the Ycf39-less mutant, this should not be the case. Indeed, overall D1 synthesis in WT and the mutant are similar (Knoppová et al. 2014) but the absence of Ycf39 makes the D1 incorporation into PSII problematic, likely due to incomplete/slow D1 folding requiring Chl binding. Nevertheless, the potential role of cyanobacterial Ycf39 in translation initiation needs to be addressed in future studies, for example using ribosome profiling. Since OHPs in plants and algae are constitutive and more essential for PSII biogenesis than HliC/D (Li et al. 2019; Wang et al. 2023), it is likely that a cyanobacterial stress-inducible route of Chl delivery to D1 via HliD/C has evolved into a constant and essential pathway in plants.

## Materials and methods

### *Synechocystis* strains

All strains used in this study were constructed in a *Synechocystis* GT-P background (Tichý et al. 2016). To prepare the *f.chlG/*Δ*chlG*/Δ*hliC* strain, the *f.chlG/*Δ*chlG* strain described in (Chidgey et al. 2014) was transformed with genomic DNA isolated from the Δ*hliC* mutant (Konert et al. 2022), and the mutated Δ*hliC* locus fully segregated by plating on an increasing concentration of chloramphenicol. The resulting strain was subsequently transformed using genomic DNA from the Δ*ycf39* mutant (Knoppová et al. 2014) to obtain the *f.chlG/*Δ*chlG*/Δ*hliC*/Δ*ycf39* strain.

The *his-hliD* strain, expressing the *8xhis-hliD* gene from the native *hliD* locus, was prepared using a synthetic DNA construct (GenScript, Piscataway, USA). This construct was comprised of the *his*-*hliD* gene flanked by NdeI and BglII restriction sites, followed by a spectinomycin resistance cassette (Sp^R^). This region was surrounded with flanking 500 bp sequences corresponding to those upstream and downstream of the *hliD* gene in the *Synechocystis* genome to permit replacement of the native gene with the *8*x*his*-*hliD*-Sp^R^ sequence by homologous recombination. This plasmid was also used to generate another *Synechocystis* strain expressing the truncated *8xhis-*^Δ*11*^*hliD* variant using a PCR product cloned into *NdeI* and *BglII* restriction sites (see Supplementary Table S2 for primers). Both constructs were transformed into WT cells and the *hliD* locus was fully segregated on agar plates with the increasing spectinomycin concentration. For the HliC purifications, we employed the *his-hliC*/Δ*hliC* strain described in (Shukla et al. 2018), expressing 8x*his-hliC* from the *psbAII* promoter and lacking the native *hliC* gene. This mutant was transformed with genomic DNA from the Δ*hliD* mutant as described above to generate the *his*-*hliC*/Δ*hliC*/Δ*hliD* strain.

### Growth conditions

All strains were cultivated in 40 mL BG11 medium in 250-mL Erlenmeyer flasks at 28 °C with shaking and 40 *µ*mol photons m^−2^ s^−1^ (NL). At exponential phase the cultures were shifted to HL conditions (300 *µ*mol photons m^−2^ s^−1^) and harvested after a defined time, as indicated. For the purification of f.ChlG, 4 L cultures were grown to an optical density at 750 nm (OD_750_) of 1.0 to 1.5 in homemade bioreactors bubbled with sterile air at 28 °C under NL or HL illumination for 2 h. For the isolation of His-HliD and His-HliC, cells were cultivated in 1 L cylinders with 700 mL BG11, at 28 °C, NL. At an OD_750_ of 1.0 to 1.2, the expression of Hlips was induced by 500 *µ*mol of photons m^−2^ s^−1^ for 2 h before harvesting. Light intensity was measured with a LI-250A light meter with flat sensor (Li-Cor Biosciences).

### Preparation of cellular membranes

Cells were harvested by centrifugation at 8,000 × *g* at 4 °C for 10 min and either stored as pellets at −75 °C, or directly resuspended in 25 mm MES/NaOH pH 6.5, 10 mm CaCl_2_, 10 mm MgCl_2%_ and 25% (w/v) glycerol (thylakoid buffer). The cells harvested from a 40 mL culture were resuspended in 200 *µ*L of thylakoid buffer containing 1× complete protease inhibitor cocktail (Roche) in 2 mL lysis tubes and mixed with 200 *µ*L glass beads (100–200 *µ*m). The cellular membranes were extracted at 0 °C (Precellys Evolution, Bertin Technologies). Lysis program was set to 3 × 20 s at 5,500 rpm with 120 s pauses between the cycles. After cell lysis, the material was kept cold under dim green light. From the obtained lysate the glass beads were washed with the total volume of 2 mL thylakoid buffer and the cell membranes were pelleted (36,000 × *g*, 4 °C, 30 min). The supernatant was discarded, and the pellet resuspended with a metal rod in 100 *µ*L thylakoid buffer. For the preparation of membranes from 4 L of cells, the cell pellet was re-suspended in 2 mL thylakoid buffer containing 1× complete protease inhibitor cocktail (Roche) and mixed with 10 mL glass beads (100–200 *µ*m) in two 15 mL lysis tubes. The lysis program was set to 5 × 20 s at 7,500 rpm with 2 min break between the cycles and was repeated 3 times. The glass beads were washed with 30 mL of thylakoid buffer and membranes pelleted by centrifuging at 42,000 × *g*, 4 °C for 40 min. The supernatant was discarded and the pellet resuspended in 10 mL thylakoid buffer with a brush.

### Isolation of protein complexes

The membranes used for the protein pulldowns were diluted with thylakoid buffer with SIGMAFAST EDTA-free protease inhibitor (Sigma-Aldrich) to reach 0.5 mg Chl mL^−1^ and solubilized for 30 min at 10 °C with gentle rotation with a mixture of 1% (w/v) *n*-dodecyl-β-D-maltoside and 1% (w/v) glycol-diosgenin. Insoluble contaminants were removed by centrifugation (43,000 × *g*, 4 °C, 40 min). The purification of f.ChlG was carried out as described in (Koskela et al. 2020); however, a mixture of 0.02% (w/v) *n*-dodecyl-β-D-maltoside and 0.02% (w/v) glycol-diosgenin in thylakoid buffer was used instead of 0.04% (w/v) *n*-dodecyl-β-D-maltoside for all steps of the purification. The purification of His-tagged Hlips was performed essentially as described in (Konert et al. 2022).

### Protein electrophoresis and immunoblotting

Solubilized membrane proteins or isolated complexes were separated by 4% to 14% (w/v) BN-PAGE (Schagger and von Jagow 1991) or CN-PAGE with 1% (w/v) A8-35 amphipol, or in the case of radiolabelled samples, 0.05% Na deoxycholate in the running buffer according to (Kameo et al. 2021) or (Wittig et al. 2007), respectively. Individual components of protein complexes were resolved by incubating the gel strip from the first dimension in 2% (w/v) SDS and 1% (w/v) dithiothreitol for 30 min at room temperature, and proteins were separated in the second dimension by SDS–PAGE in a denaturing 16% to 20% (w/v) polyacrylamide gel containing 7 m urea (Dobáková et al. 2009). The same gel composition was used for single-dimensional SDS–PAGE.

Proteins were stained by Coomassie Brilliant Blue (CBB), or in the case of subsequent immunoblot, by SYPRO Orange Protein Gel Stain (Sigma). For the detection by a specific antibody, proteins were transferred from the SDS gel onto a polyvinylidene difluoride membrane (Immobilon-P, Merck) according to (Dobáková et al. 2009). The following primary antibodies were used in the study: anti-ChlG (Chidgey et al. 2014), anti-Ycf39 (Knoppová et al. 2014), anti-HliC antibody raised in rabbits against a synthetic peptide corresponding to unique HliC residues 1 to 17. The antibody against HliD was purchased from Agrisera (product number AS101615). The primary antibodies were probed with anti-rabbit IgG-peroxidase antibody produced in goat (Sigma-Aldrich, A6154) and visualized using Immobilon Crescendo Western HRP substrate (Millipore) and luminescence image analyzer (ImageQuant, LAS-4000).

### Protein radiolabeling

Cells were incubated with a mixture of [^35^S]-Met and [^35^S]-Cys (Hartmann Analytics) at NL or 500 *µ*mol photons m^−2^ s^−1^ for 20 min, as described in (Dobáková et al. 2009). Cellular membranes were isolated and separated by 2D CN/SDS-PAGE using Na deoxycholate in the running buffer as described above. The 2D gel was stained by CBB, photographed, dried, exposed to a phosphorimager plate (GE Healthcare) overnight, and scanned by Storm 860 (GE Healthcare).

### Modeling the interactions between *Ycf39*, *ChlG*, and *HliD*

A model structure was constructed for the G-D_2_-39 complex containing 4 Chls using AlphaFold3 (Abramson et al. 2024 ). This structure was then used as the input to paramaterization and membrane embedding using the CHARMM-GUI server (Jo et al. 2008). Orientation of the molecule in the membrane was defined with the PPM 2.0 web server (Lomize et al. 2012). The lipid bilayer is modeled as 74% monogalactosyldiacylglycerol and 26% phosphatidylglycerol. This simplified lipid composition, lacking digalactosyldiacylglycerol and sulfoquinovosyldiacylglycerol, is to eliminate noise from potential interactions between Ycf39 and the larger head groups of those lipids. Potassium counter ions were added to neutralize system.

MD simulation was run for 1 µs, specifying a constant number of particles, temperature and pressure with GROMACS 2021.4 and CHARMM36 force field (Best et al. 2012). The Nosé-Hoover algorithm was used for temperature coupling and the Parrinello-Rahman algorithm for constant pressure under semi-isotropic periodic boundary conditions. Stability of the complex was determined by the RMSD of C-α atoms. Interactions formed between proteins in a complex were analysed using the GetContacts tool (https://getcontacts.github.io). MD simulation trajectory files were visualized by ChimeraX (Meng et al. 2023). Movies were generated using PyMOL 3.0 (The PyMOL Molecular Graphics System, Schrödinger, LLC).

### Phylogenetic analysis of HliC and HliD

To understand conservation of cyanobacterial HliC and HliD residues their homologs were retrieved from 37 evolutionarily representative cyanobacteria selected based on their relationships in multigene phylogenies. Predicted proteins of the 37 species were downloaded from NCBI Datasets and searched individually by BLASTP at the *e*-value cutoff of 1 × 10^−3^. Homologous sequences, defined by the presence of the Chl-binding domain, were subsequently aligned in MAFFT v7.505 (localpair algorithm; Katoh and Standley 2013). Sequences orthologous to the reference HliC and HliD from *Synechocystis* were identified by a maximum likelihood phylogeny inferred in IQ-TREE v.2.3.6, which included best-fit model selection in ModelFinder (MFP setting; Minh et al. 2020). Nonorthologous and fast-evolving sequences were removed, and the modified HliC and HliD alignments were used to construct sequence logos in Jalview 2.11 (Waterhouse et al. 2009).

### Single particle electron microscopy analysis

Single-particle EM of specific forms of f.ChlG protein complexes was performed after their extraction from the gel. The excised bands were cut into small pieces and extracted with thylakoid buffer at 4 °C overnight according to (Kouřil et al. 2014), which additionally contained critical micelle concentration of n-dodecyl-β-D-maltoside. The supernatants were directly used for grid preparation. Samples were prepared on glow-discharged carbon coated copper grids and negatively stained with 2% uranyl acetate. Electron micrographs were collected using a Tecnai G2 F20 microscope (FEI Technologies, Hillsboro, USA) with an Eagle 4 K CCD camera (FEI Technologies, Hillsboro, USA). Electron micrographs were recorded at 134,028× magnification. The pixel size at the specimen level after binning the images to 2,048 × 2,048 pixels was 0.224 nm. Approximately 200,000, 255,000, and 40,000 projections were picked in semi-automated mode from 4,600, 5,400, and 6,200 micrographs of specimens prepared from the gel bands assigned as G-D_2_-G, G-D_2_-39 and RCCII-Hlips-ChlG, respectively. Individual datasets were subjected to reference free 2-dimensional classification using SCIPION image processing framework (de la Rosa-Trevin et al. 2016). AlphaFold3 structures of G-D_2_-G and RCCII-Hlips-f.ChlG (Fig. 4) and various snapshots from MD of G-D_2_-39 complexes (Supplementary Fig. S7) were used to fit the projection maps of analyzed protein complexes.

### Quantitative protein mass-spectrometry

The eluate from the immobilized anti-FLAG antibody resin was mixed with 30 pmol of an ^15^N-labeled internal standard in the form of an artificial protein sequence (Pratt et al. 2006). This protein contained concatenated proteotypic tryptic peptide sequences belonging to ChlG, HliD and Ycf39 (see Supplementary Table S1) and was expressed in *E. coli* as described previously (Qian et al. 2013). The mixed immuno-captured and ^15^N-labeled standard proteins were precipitated using a 2D clean-up kit (Cytiva) and dissolved in 8 m urea, 100 mm Tris-HCl pH 8.5. Subsequent processing, including digestion with a combination of endoproteinase Lys-C and trypsin followed by analysis using nano-flow liquid chromatography coupled to MS, was carried out according to (Hitchcock et al. 2016). MS data-files were converted to mzML format using ProteoWizard (Chambers et al. 2012) prior to database searching to identify proteotypic peptides, both ^14^N from the FLAG eluates and ^15^N from the internal standard. The database used was the *Synechocystis* sp. PCC 6803 reference proteome database (https://www.uniprot.org/proteomes/UP000001425) downloaded on October 18 2024. The search engine was Mascot Daemon v. 2.5.1 running with Mascot Server v. 2.5.1 (Matrix Science). Picomolar amounts of f.ChlG, HliD and Ycf39 were calculated from the relative intensities of the ^14^N and ^15^N peptide monoisotopic ions extracted from the MS data-files using the Qual Browser application in Xcalibur v. 4.2.47 (Thermo Fisher Scientific).

### Analysis of pigments by HPLC

To analyse the pigments from protein complexes, specific bands of CN gels containing respective protein complexes were excised, and the pigments were extracted and detected according to (Konert et al. 2022).

### Accession numbers

Sequence data from this article can be found in the GenBank/EMBL data libraries under accession numbers: HliD, P72932.1; HliC, P73563.2; ChlG, BAA10281.1; Ycf39, AGF52218.1.

## Supplementary Material

kiaf213_Supplementary_Data

## Data Availability

The original contributions presented in the study are included in the article and the associated Supporting Information. The mass spectrometry proteomics data have been deposited to the ProteomeXchange Consortium (http://proteomecentral.proteomexchange.org) via the PRIDE partner repository with the dataset identifier PXD058942.

## Supplementary data

The following materials are available in the online version of this article.

**Supplementary Table S1.** Proteotypic tryptic peptide sequences used to construct the artificial ^15^N-labeled internal standard protein.

**Supplementary Table S2.** The primer sequences used in this study.

**Supplementary Figure S1.** Accumulation of *Synechocystis* ChlG, Ycf39, HliD, and HliC proteins during HL stress conditions.

**Supplementary Figure S2.** 2D CN/SDS-PAGE of the f.ChlG eluates and the control WT pulldown.

**Supplementary Figure S3.** 2D BN/SDS-PAGE and immunodetection of (f.)ChlG and Ycf39 in thylakoid membranes isolated from Δ*hliC* and *f.chlG*/Δ*chlG*/Δ*hliC* cells.

**Supplementary Figure S4.** AlphaFold3 models of the selected *Synechocystis* ChlG complexes.

**Supplementary Figure S5.** A gallery of classified single particle images of protein complexes eluted from CN-PAGE gel band representing the G-D_2_-G complex.

**Supplementary Figure S6.** A gallery of classified single particle images of protein complexes representing the purified G-D_2_-39 assembly.

**Supplementary Figure S7.** Analysis of MD simulation of the G-D_2_-39 complex.

**Supplementary Figure S8.** Fitting of representative G-D_2_-39 structures from MD simulations to the electron microscopy images.

**Supplementary Figure S9.** A gallery of classified single particle images of protein complexes representing the purified RCCII-Hlips-ChlG assembly.

**Supplementary Figure S10.** Structural models of the *Synechocystis* 39-D/C and the homologous Arabidopsis YCF244-OHP1-OHP2 complexes.

**Supplementary Data Set 1.** Extracted ion counts for the protein MS quantification.

**Supplementary Video S1.** MD simulation of the G-D_2_-39 complex embedded in thylakoid membrane—overview of the system.

**Supplementary Video S2.** MD simulation of the G-D_2_-39 complex embedded in thylakoid membrane—run 2.

## Dive Curated Terms

The following phenotypic, genotypic, and functional terms are of significance to the work described in this paper:

OHP2 Gramene: AT1G34000
OHP2 Araport: AT1G34000

NCBI Gramene: https://www.ncbi.nlm.nih.gov
NCBI Araport: https://www.ncbi.nlm.nih.gov

PCR Gramene: Polymerase Chain Reaction
PCR Araport: Polymerase Chain Reaction

ChlG Gramene: AT3G51820
ChlG Araport: AT3G51820
