# Supplementary material for: High-light-inducible proteins control associations between chlorophyll synthase and the Photosystem II biogenesis factor Ycf39
Source: Plant Physiol. 2025 May 25;198(2):kiaf213. doi: 10.1093/plphys/kiaf213 (PMC12142464; doi:10.1093/plphys/kiaf213)
Supplement: kiaf213_Supplementary_Data [file kiaf213_supplementary_data.zip › Supplementary data.pdf]

SUPPLEMENTARY DATA

Supplementary information includes two supplementary tables, ten figures, one supplementary dataset, two supplementary videos and supplementary video legends.

Supplementary Table S1. **Proteotypic tryptic peptide sequences used to construct the artificial ^{15}N-labeled internal standard protein**.

The sequence elements were incorporated into the ^{15}N-labelled artificial internal standard protein in the order specified. Proteotypic peptides that were used in the quantification of the target proteins are highlighted in bold. Peptides were selected on the basis of their frequent identification in proteomic analyses of *Synechocystis* thylakoid membranes. N- and C-side flanking sequences were included where indicated to mimic the cleavage sites in the natural protein. Although selection of ≥ 2 peptides per protein is advisable, in the case of ChlG only GAA-IWK is consistently detectable. Additional peptides not relevant to this study were also present, making a total molecular mass of 40,963 Da for this artificial protein.

Sequence element	Tryptic peptide	Notes
pET-14b N-terminal extension	MGSSHHHHHHSSGLVPRGSH	His-tag for purification by immobilized Ni^{2+} ion affinity chromatography
2 x W insertion	AWSWK	Additional Trp-containing sequence to increase A_{280} for protein concentration measurement
ChlG spacer	LGMK	N-side flanking sequence to GAA-IWK
**ChlG-1**	**GAAPGESSIWK**	Peptide used for quantification
ChlG spacer	IRLQ	C-side flanking sequence to GAA-IWK
ChlG spacer	YFLR	N-side flanking sequence to NPL-DVK
ChlG-2	NPLENDVK	Peptide with low recovery not used for quantification
**HliD-1**	**SEELQPNQTPVQEDPK**	Peptide used for quantification
**HliD-2**	**FGFNNYAEK**	Peptide used for quantification
HliD spacer	LNGR	C-side flanking sequence to FGF-AEK

Ycf39 spacer	MR	N-side flanking sequence to VLV-LGR
**Ycf39-1**	**VLVVGGTGTLGR**	Peptide used for quantification
Ycf39 spacer	QIVR	C-side flanking sequence to VLV-LGR
Ycf39 spacer	FAVR	N-side flanking sequence to AVE-VAR
**Ycf39-2**	**AVELDSVAR**	Peptide used for quantification
Ycf39 spacer	KTYP	C-side flanking sequence to AVE-VAR
Ycf39 spacer	ASDR	N-side flanking sequence to LAF-SGK
Ycf39-3	LAFSEVLASGK	Peptide with low recovery not used for quantification
Ycf39 spacer	ALTA	C-side flanking sequence to LAF-SGK

Supplementary Table S2. **The primer sequences used in this study.**

**Primer**	**Primer sequence**
His-hliD_f	TAATAACATATGCACCATCACCATCACCATCACCATGGAAGTGAAGAACTACAACCG-3'
His-hliD_r	GCAATAAGCCAGATCTCTAGCGCAGTCCCAACCAGG-3'
His-Δ1-11-hliD_f	TCGAACATATGCACCATCACCATCACCATCACCATGGAGTGCAGGAAGATCCCAAATTTGG-3'
His-Δ1-11-hliD_r	ATGCTAGATCTCTAGCGCAGTCCCAACCAG-3'

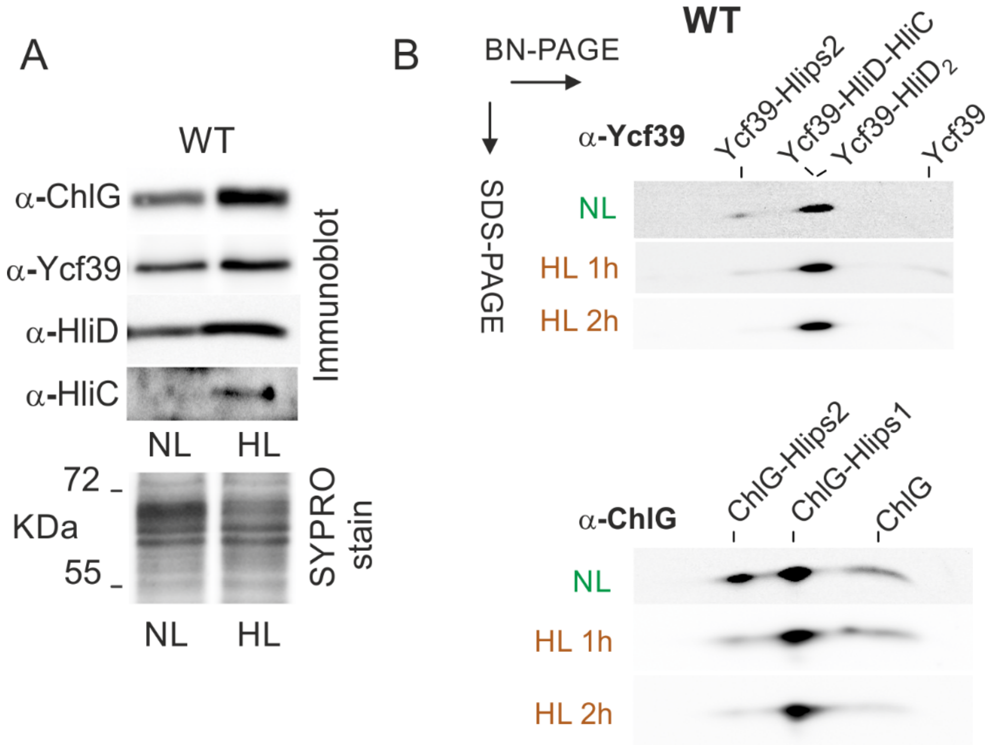

Supplementary Figure S1. Accumulation of *Synechocystis* ChlG, Ycf39, HliD and HliC proteins during normal and high light conditions (NL, HL). (**A**) Membrane proteins from WT grown under NL or after 16h at HL were separated by SDS-PAGE, blotted and sequentially probed using the indicated antibodies. A part of the SYPRO Orange stained gel is shown as loading controls. (**B**) *Synechocystis* WT cells were grown under NL conditions and then shifted to HL for either 1 h or 2 h. Membrane proteins isolated from these cell cultures were separated by 2D BN/SDS-PAGE and analyzed by immunoblotting using antibodies against ChlG and Ycf39 (α-ChlG and α-Ycf39). The separate segments of the blot with individual antibody signals are shown (see Figure 2, main text).

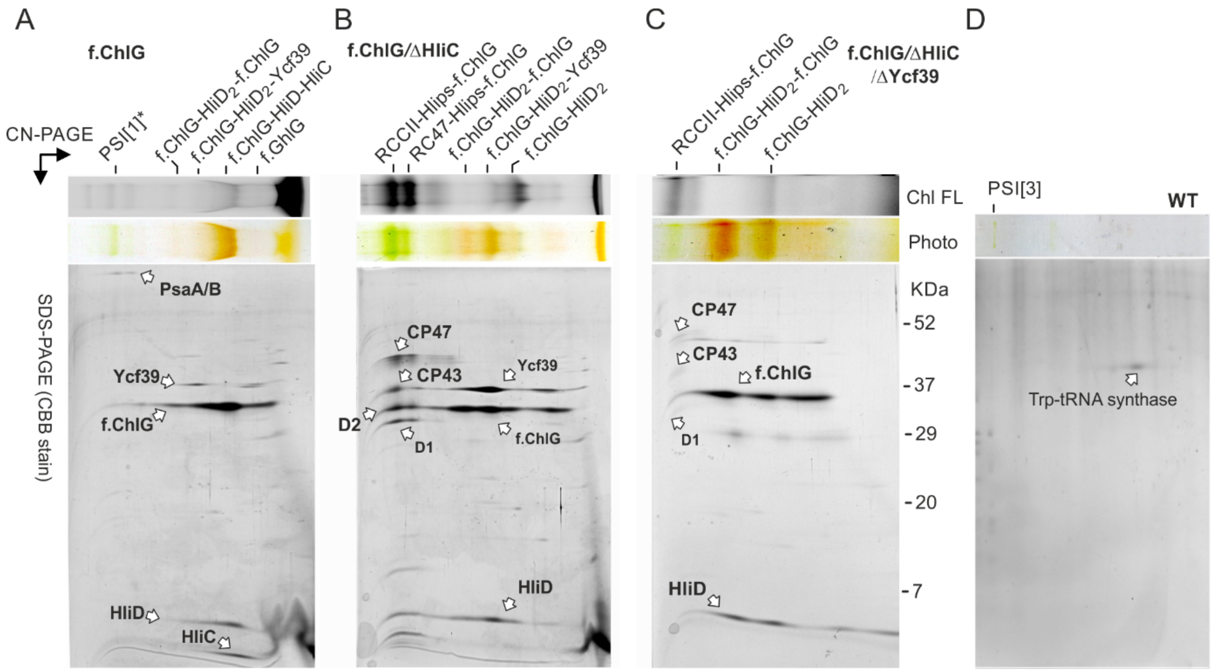

Supplementary Figure S2. Co-immunopurification of ChlG.

2D CN/SDS-PAGE analysis of co-immunopurified protein from membrane isolates derived from the **(A)** *f.chlG*/Δ*chlG*, **(B)** *f.chlG*/Δ*chlG*/Δ*hliC*, **(C)** *f.chlG*/Δ*chlG*/Δ*hliC*/Δ*ycf39*, and **(D)** WT strains. The gel was photographed (Photo) and scanned by LAS 4000 (FUJI) for Chl fluorescence (Chl FL). The protein complexes resolved by CN-PAGE were further separated in a second dimension by SDS-PAGE and stained with Coomassie Brilliant Blue (CBB stain). Proteins indicated by arrows on the stained gels were assigned according to (Chidgey et al., 2014; Knoppová et al., 2014); tryptophanyl-tRNA synthetase (Slr1884) is a known non-specific interactor with the FLAG resin (Knoppová et al., 2014). The PsaA/B spots (A) are derived from PSI[1]* which is an uncharacterized form of monomeric PSI that co-isolates with f.ChlG (Chidgey et al., 2014).

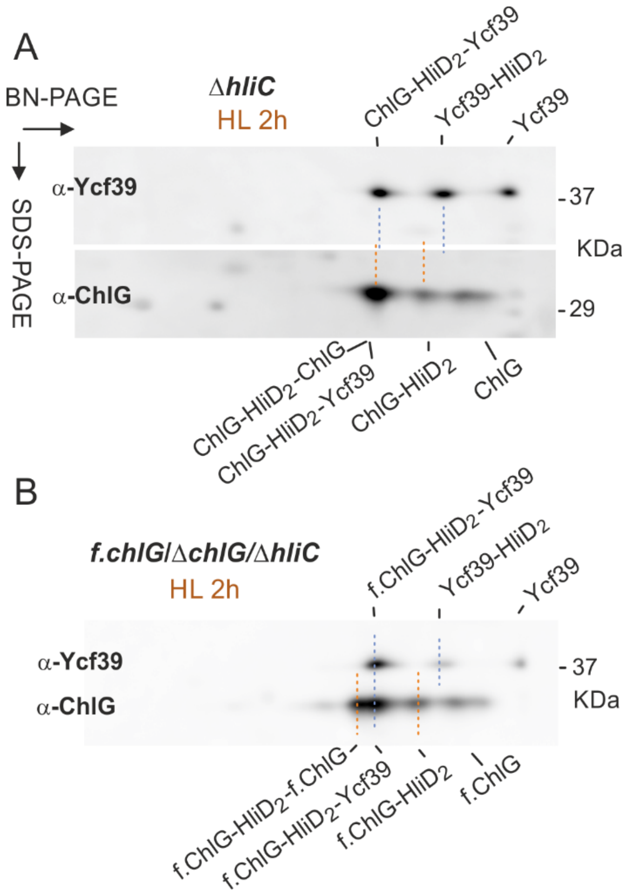

Supplementary Figure S3. 2D BN/SDS-PAGE and immunodetection of (f.)ChlG and Ycf39 in thylakoid membranes isolated from Δ*hliC* and *f.chlG*/Δ*chlG*/Δ*hliC* cells. (A) *Synechocystis* Δ*hliC* cells were grown under NL conditions and then shifted to HL for 2 h. Membrane proteins isolated from these cell cultures were separated by 2D BN/SDS-PAGE and analyzed by immunoblotting with ChlG and Ycf39 antibodies. The separate segments of the blot with individual antibody signals are shown. Dotted blue lines indicate 39-D_2 and G-D_2-39 complexes (see also Figure 2C). Dotted orange lines indicate G-D_2 and G-D_2-G. Note that the G-D_2 and G-D_2-39 show very similar mobility on the BN gel. **(B)** The identical separation of thylakoid membranes isolated from the *f.chlG*/Δ*chlG*/Δ*hliC* strain after 2h at HL300. As the 3xFLAG tag affects the mobility of f.ChlG in the BN gel, the G-D_2-G and G-D_2-39 complexes can be distinguished.

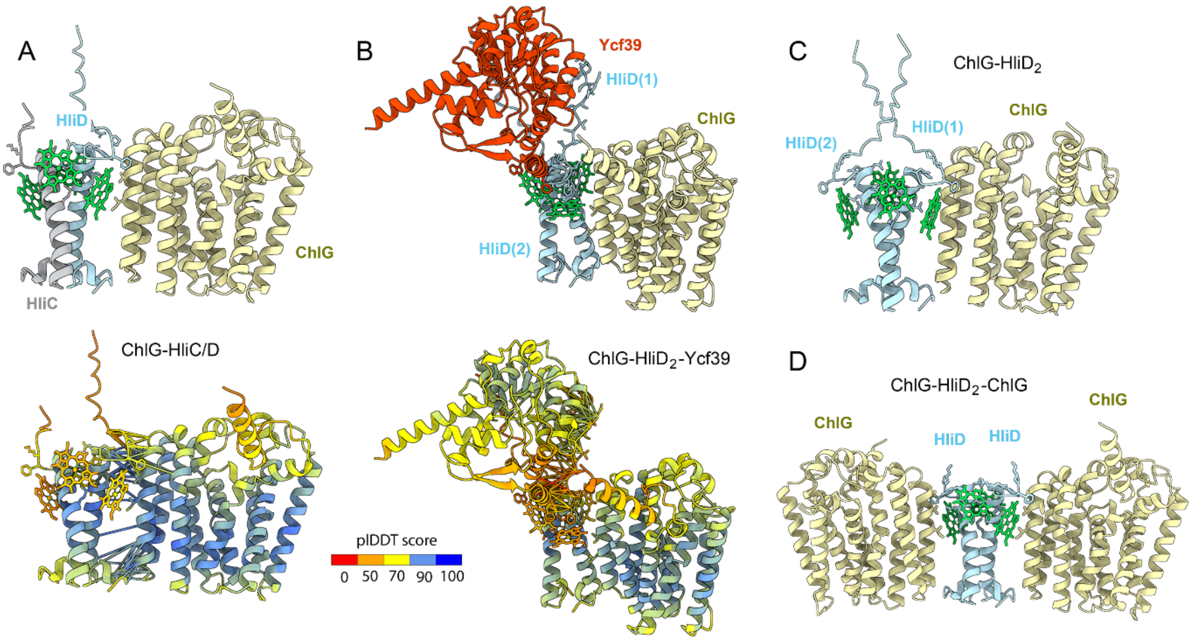

Supplementary Figure S4. AlphaFold3 models of the selected *Synechocystis* ChlG complexes. **(A)** A model G-D/C complex with 4 Chls as cofactors. The lower image shows the predicted local distance difference test (plDDT) scores for all subunits and contacts (up to 7Å) between ChlG and HliD(1). The plDDT score indicates the confidence that a structure is predicted accurately. **(B)** The same calculation as in (A) for the G-D$_2$-39 complex; the plDDT score includes contacts (up to 7Å) between Ycf39 and HliD(1). The flexible, C-terminal segment of Ycf39, is omitted for the clarity. (**C, D**) AlphaFold3 models for G-D$_2$ and G-D$_2$-G complexes respectively. Images were prepared in ChimeraX (Meng et al., 2023).

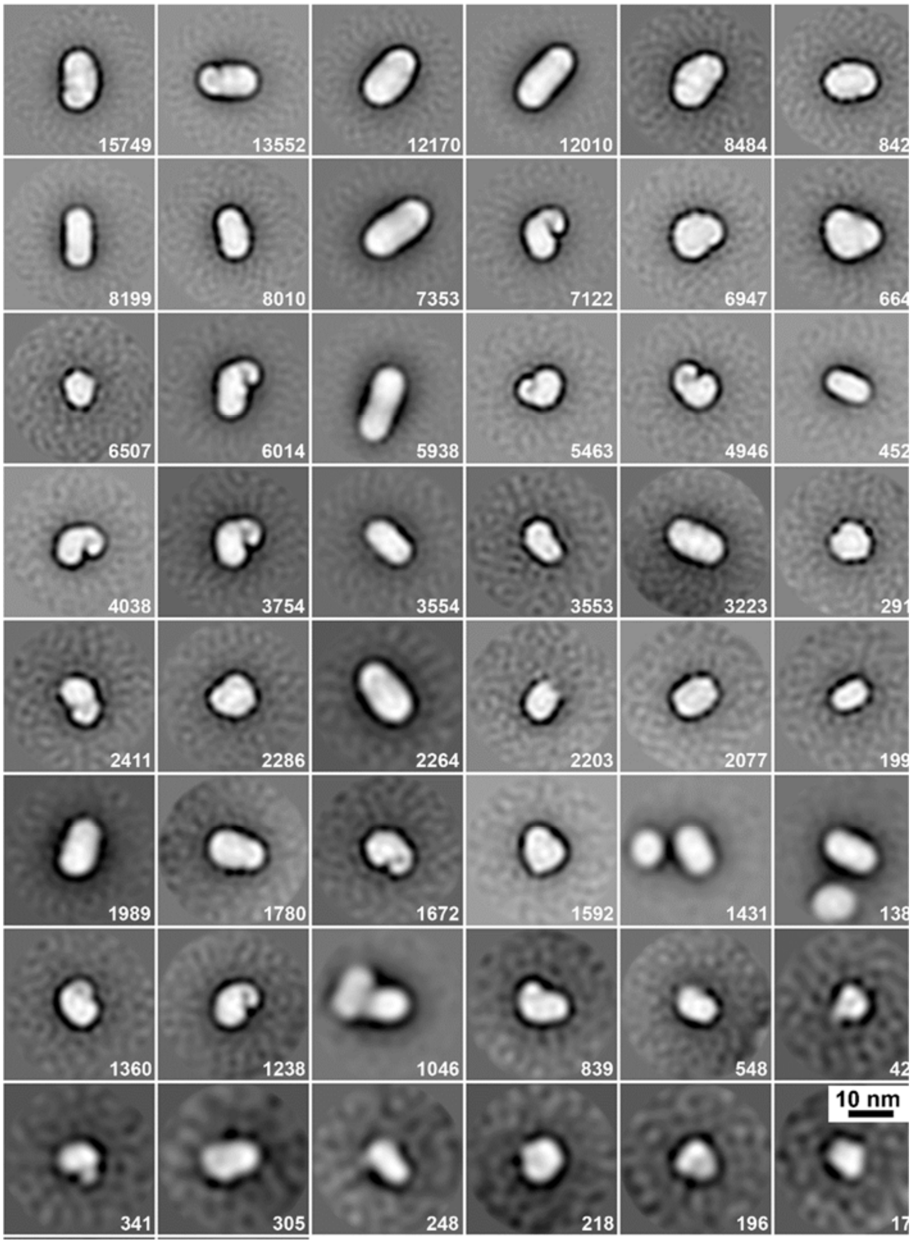

Supplementary Figure S5. **A gallery of classified single particle images of protein complexes eluted from CN-PAGE gel band representing the G-D_2-G complex.** Single particle EM analysis of proteins, eluted from the CN-PAGE band that was assigned as the G-D_2-G assembly (see Figure 3, main text). The cut piece of CN gel was incubated overnight in elution buffer containing 50 mM HEPES pH 7.2, 5 mM $MgCl_2$, 15 mM NaCl and critical micelle concentration (CMC) of *n*-dodecyl-β-D-maltoside. The number of particles averaged for each class is shown in the bottom right corners; the scale bar is same for all images.

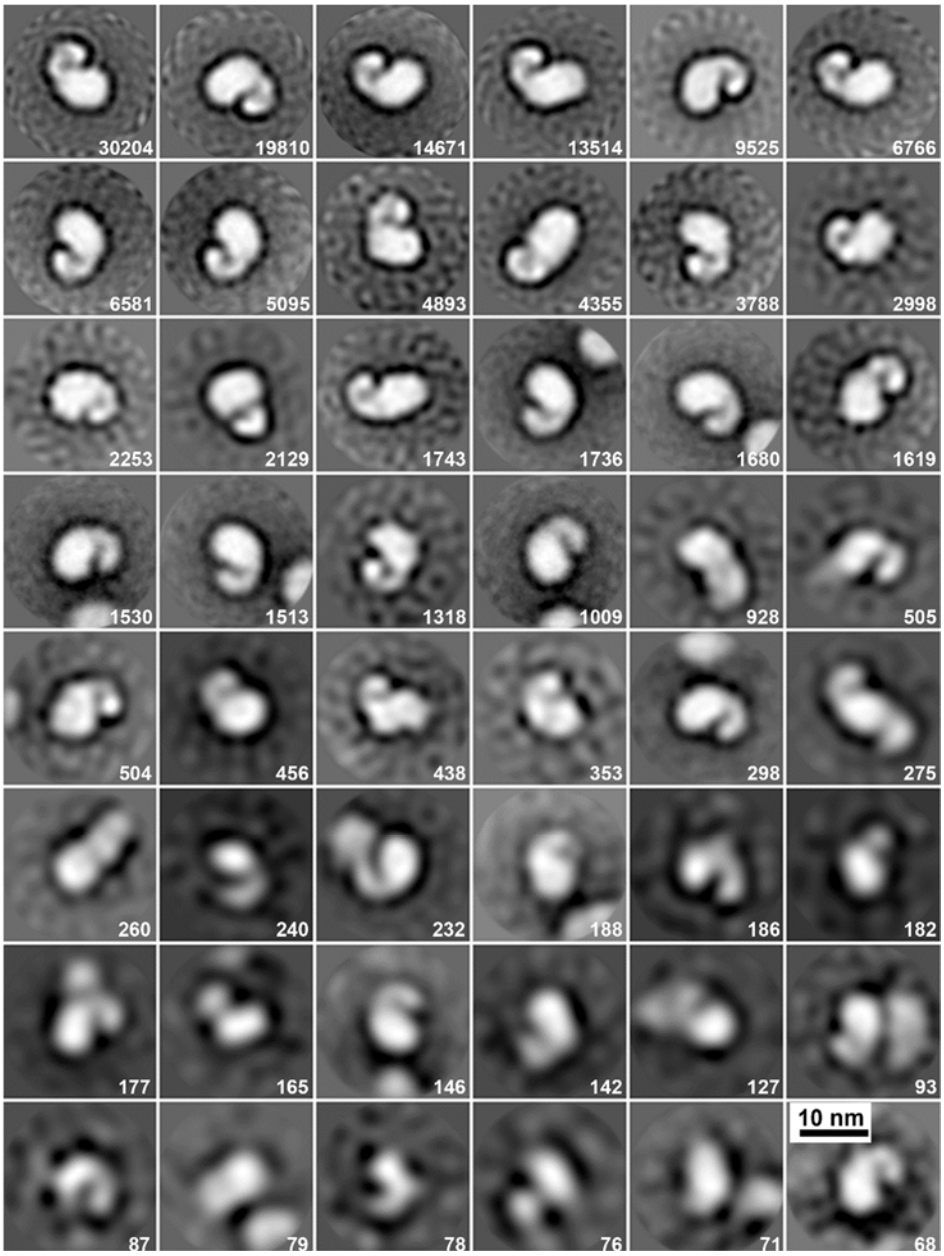

Supplementary Figure S6. **A gallery of classified single particle images of protein complexes representing the purified G-D$_2$-39 assembly.** Single particle EM analysis of proteins, eluted from the CN-PAGE band that was assigned as the G-D$_2$-39 assembly (see Figure 3 in main text). The protein sample was prepared as described in Supplementary Figure S5; the number of particles averaged for each class is shown in the bottom right corners. The scale bar is same for all images.

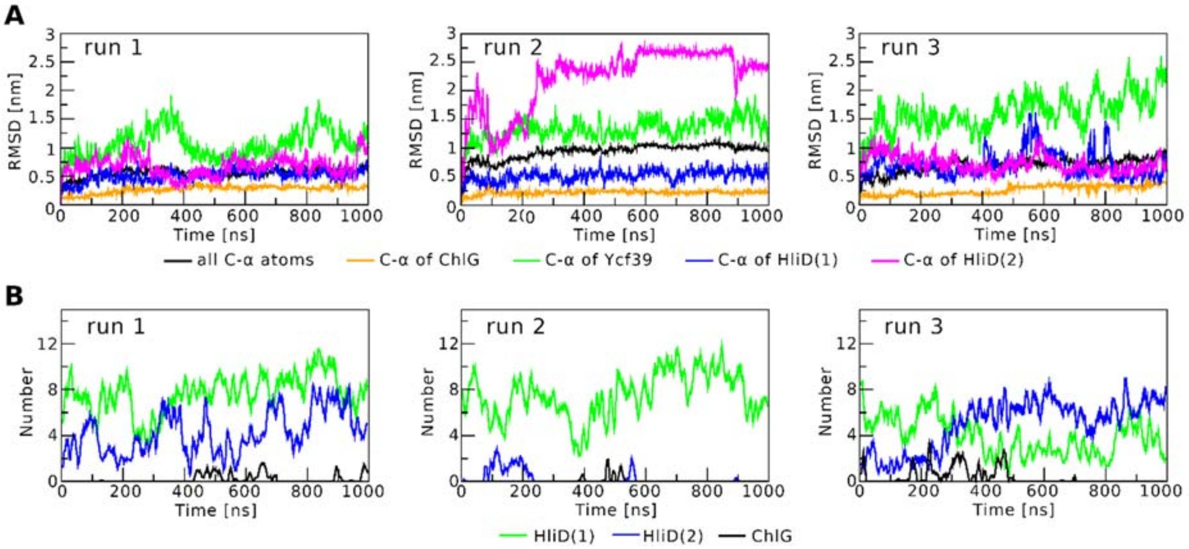

Supplementary Figure S7. Analysis of MD simulation of the G-D_2-39 complex. **(A)** RMSD of C-alpha atoms of ChlG (orange), HliD(1) (blue), HliD(2) (magenta) and Ycf39 (green). Fitting of the complexes for calculation was done in respect to the ChlG C-alpha atoms. RMSD calculation for all system C-alpha atoms fitted to the initial structure of itself (full complex) is shown in black. See Supplemental Fig. 4 for the description of G-D_2-39 and the identity of HliD(1) and HliD(2). **(B)** Number of hydrogen bonds formed between Ycf39 and other proteins (data are averaged over 10 snapshots), calculated using GROMACS.

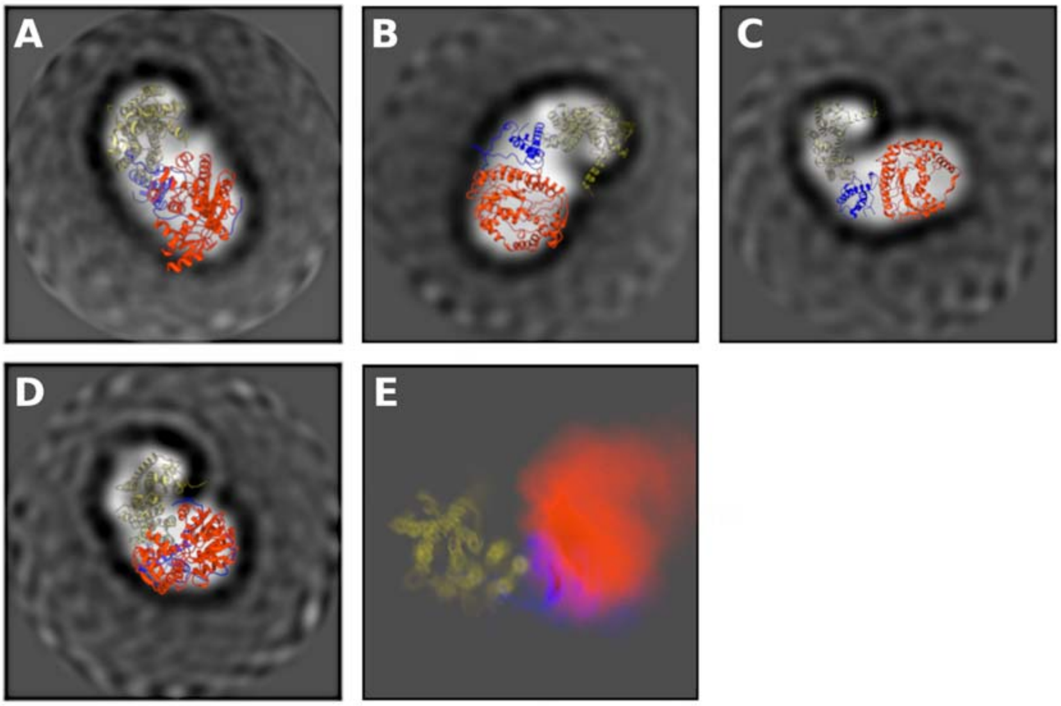

Supplementary Figure S8. **The flexibility of Ycf39 in the G-D$_2$-39 structure. (A-D)** Colours are: ChlG - khaki, HliD – blue, Ycf39 – orange red (representative structures are from cluster analysis). Please note that the microscopy images in panels A and D are the same as those shown in Figure 4B. (**E**) Density map of the Ycf39 - orange red; HliD – blue; ChlG - khaki. The view is from the one side of the membrane for all snapshots from 3 MD simulation runs (see Supplementary Figure S7). Backbone atoms only were used for visualization. The well-resolved structure and intensely coloured density map of ChlG indicates a relatively stable position of this protein in a membrane, while Ycf39 is smeared and we could not recognize individual secondary structure elements, indicating high flexibility. The size of the red area shows the scope of the motion of Ycf39 in respect to ChlG. The more intense color means higher frequency of the presence of backbone atom at some point over the MD simulation. Analysis was done with GROMACS and structural visualization with ChimeraX (Meng et al., 2023).

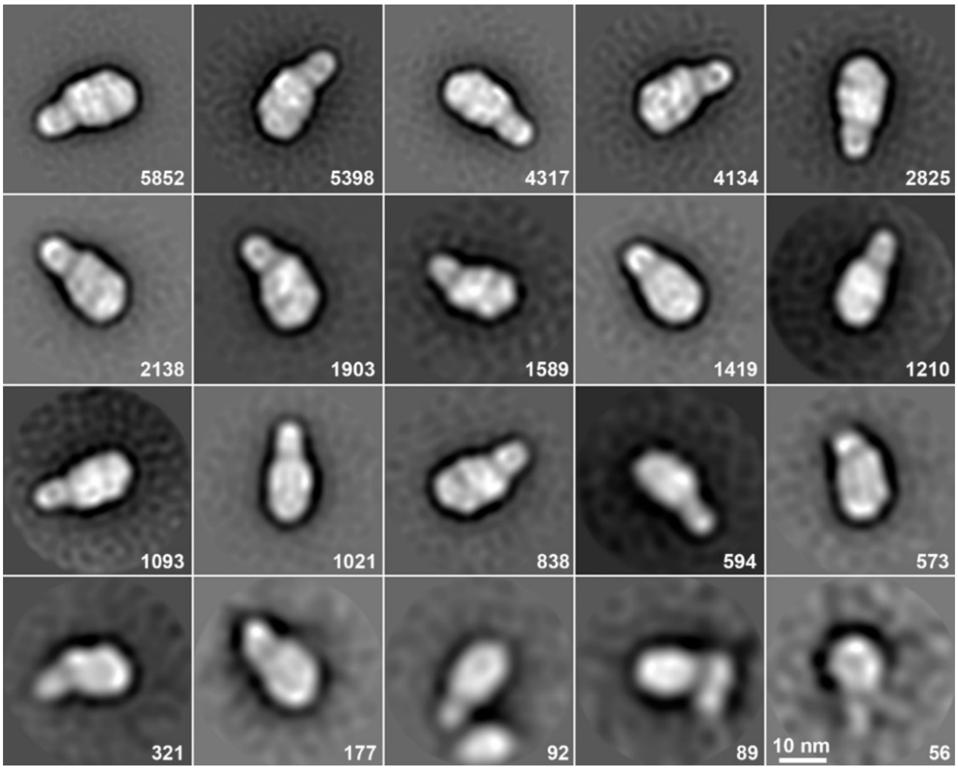

Supplementary Figure S9. **A gallery of classified single particle images of protein complexes representing the purified RCCII-Hlips-ChlG assembly**. Single particle EM analysis of proteins, eluted from the CN-PAGE band that was assigned as the RCCII-Hlips-ChlG assembly (see Figure 3, main text). The protein sample was prepared as described in Supplementary Figure S5; the number of particles averaged for each class is shown in the bottom right corners. the scale bar is same for all images.

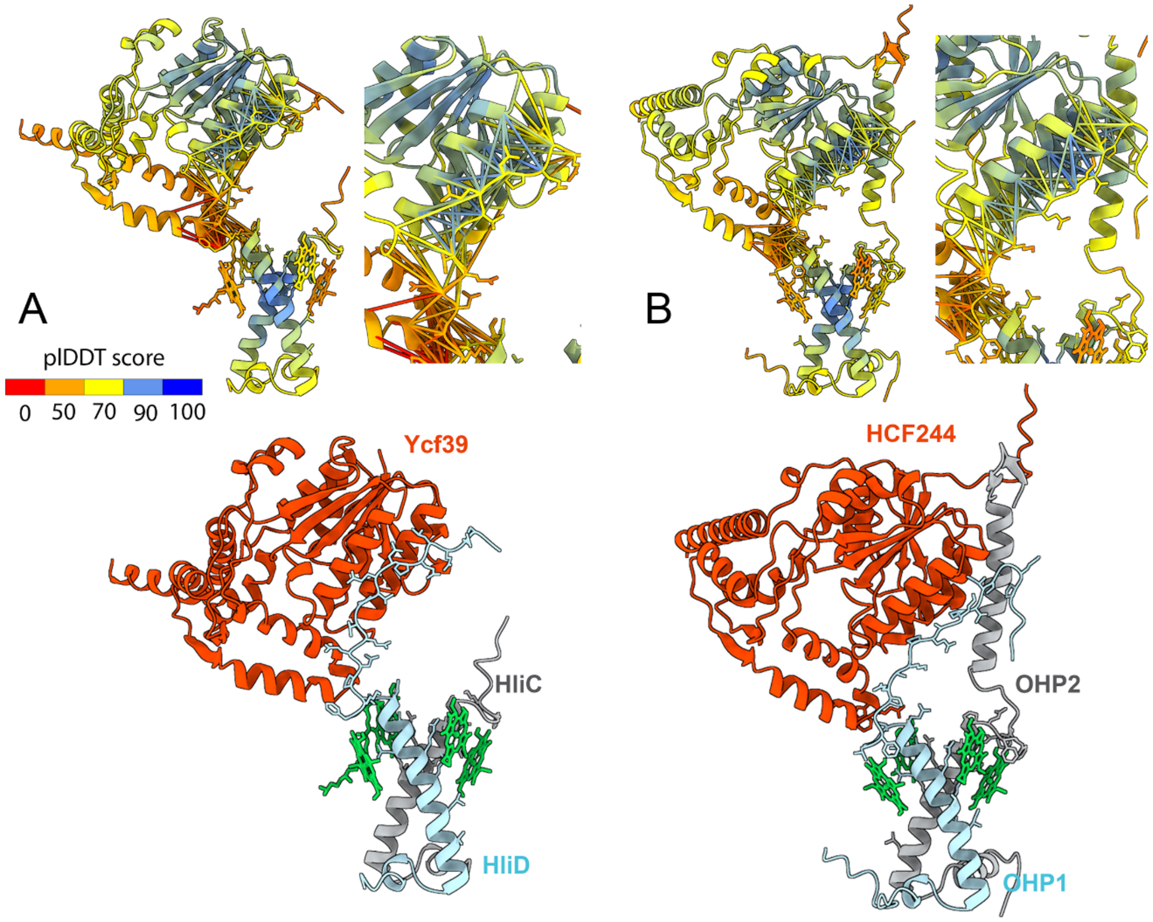

Supplementary Figure S10. Structural models of the *Synechocystis* 39-D/C (A) and the homologous Arabidopsis HCF244-OHP1-OHP2 (B) complexes. The models were predicted by AlphaFold3 (Abramson et al., 2024) and the calculation included four Chl molecules as cofactors. The top part of each panel shows the plDDT score (predicted local distance difference test) for all subunits, in a detailed view, for contacts (up to 7Å) between HliD/OHP1 N-terminus and Ycf39/HCF244. Higher score indicates higher confidence and usually a more accurate prediction. Images were prepared using ChimeraX (Meng et al., 2023).

References

Abramson J, Adler J, Dunger J, Evans R, Green T, Pritzel A, Ronneberger O, Willmore L, Ballard AJ, Bambrick J, Bodenstein SW, Evans DA, Hung CC, O'Neill M, Reiman D, Tunyasuvunakool K, Wu Z, Zemgulyte A, Arvaniti E, Beattie C, Bertolli O, Bridgland A, Cherepanov A, Congreve M, Cowen-Rivers AI, Cowie A, Figurnov M, Fuchs FB, Gladman H, Jain R, Khan YA, Low CMR, Perlin K, Potapenko A, Savy P, Singh S, Stecula A, Thillaisundaram A, Tong C, Yakneen S, Zhong ED, Zielinski M, Zidek A, Bapst V, Kohli P, Jaderberg M, Hassabis D, Jumper JM (2024) Accurate structure prediction of biomolecular interactions with AlphaFold 3. Nature **630:** 493-500

Chidgey JW, Linhartová M, Komenda J, Jackson PJ, Dickman MJ, Canniffe DP, Koník P, Pilný J, Hunter CN, Sobotka R (2014) A cyanobacterial chlorophyll synthase-HliD complex associates with the Ycf39 protein and the YidC/Alb3 insertase. Plant Cell **26:** 1267-1279

Knoppová J, Sobotka R, Tichý M, Yu J, Koník P, Halada P, Nixon PJ, Komenda J (2014) Discovery of a chlorophyll binding protein complex involved in the early steps of photosystem II assembly in *Synechocystis*. Plant Cell **26:** 1200-1212

Meng EC, Goddard TD, Pettersen EF, Couch GS, Pearson ZJ, Morris JH, Ferrin TE (2023) UCSF ChimeraX: Tools for structure building and analysis. Protein Sci. **32:** e4792
