# Supplementary material for: High-light-inducible proteins control associations between chlorophyll synthase and the Photosystem II biogenesis factor Ycf39
Source: Plant Physiol. 2025 May 25;198(2):kiaf213. doi: 10.1093/plphys/kiaf213 (PMC12142464; doi:10.1093/plphys/kiaf213)
Supplement: kiaf213_Supplementary_Data [file kiaf213_supplementary_data.zip › Supplementary_Video_Legends.docx]

**Supplementary Video Legends**

**Supplementary Video S1. MD simulation of the G-D_2_-39 complex embedded in thylakoid membrane – overview of the system.**

The video shows the system used for the MD simulation: the top of the membrane (from the cytoplasm) and side view. Water molecule, neutralizing ions, hydrogens are hidden for clarity. Protein molecules are shown as cartoons: ChlG(G) is colored yellow, HliD(1) and HliD(2) are red and green respectively, Ycf39 is blue. Chl molecules are represented by thick purple sticks. Lipid residues are represented by sticks with element color scheme (carbons - cyan, oxygens - red, phosphorus – orange). At the end of the video the color scheme for lipids is changed to illustrate the distribution of lipids as follows: monogalactosyldiacylglycerol - green, phosphatidylglycerol – orange.

**Supplementary Video S2. MD simulation of the G-D_2_-39 complex embedded in thylakoid**

Representative MD simulation of a protein complex is shown (from 0 ns to 1000 ns). Protein molecules are shown as cartoons: ChlG(G) is colored yellow, HliD(1) and HliD(2) are red and green respectively, Ycf39 is blue. Amino acid residues of Ycf39 and HliD(1) involved in hydrogen bond interaction between subunits are shown as sticks, hydrogens are hidden for clarity. Hydrogen bonds are shown as dashed lines and colored yellow. The interactions between Ycf39 and HliD(1) are zooming-in at the beginning and end of the MD simulation.
